# Harmonic patterns embedded in ictal EEG signals in focal epilepsy: new insight into the epileptogenic zone

**DOI:** 10.1186/s12916-026-04665-7

**Published:** 2026-01-28

**Authors:** Lingli Hu, Lingqi Ye, Hongyi Ye, Xiaochen Liu, Kai Xiong, Yuanming Zhang, Zhe Zheng, Hongjie Jiang, Cong Chen, Chunhong Shen, Zhongjin Wang, Jiping Zhou, Yingcai Wu, Kejie Huang, Junming Zhu, Zhong Chen, Meiping Ding, Shennan Weiss, Dongping Yang, Shuang Wang

**Affiliations:** 1 Department of Neurology and Epilepsy Center, School of Medicine, Second Affiliated Hospital, Zhejiang University, Hangzhou, 310009 China; 2 Nanhu Brain-Computer Interface Institute, Hangzhou, China; 3 College of Information Science and Electronic Engineering, Zhejiang University, Hangzhou, China; 4 College of Computer Science and Technology, Zhejiang University, Hangzhou, China; 5 Department of Neurosurgery, School of Medicine, Second Affiliated Hospital, Zhejiang University, Hangzhou, China; 6 Department of Neurology, Wayne State University/Detroit Medical Center, Detroit, MI USA; 7 Key Laboratory of Neuropharmacology and Translational Medicine of Zhejiang Province, School of Pharmaceutical Sciences, Zhejiang Chinese Medical University, Hangzhou, China; 8 Department of Neurology, Stony Brook University, Stony Brook, USA; 9 Shenzhen Loop Area Institute, Shenzhen, 518045 China

**Keywords:** Harmonic pattern, Ictal EEG, Epileptogenic zone, Nonlinear phenomena, Epilepsy surgery

## Abstract

**Background:**

Localization of the epileptogenic zone (EZ) requires further refinement. We identified a unique ictal spectral structure, the “harmonic pattern” (H pattern), which potentially serves as a novel biomarker for localizing the EZ. This study aimed to analyze the clinical significance of the H pattern and to explore its underlying waveform features.

**Methods:**

Seventy patients with drug-resistant focal epilepsy, undergoing stereo-EEG (SEEG) evaluation and surgery, were included. Time–frequency maps (TFM) were generated using Morlet wavelet transform analysis. The H pattern was defined as multiple equidistant, high-density bands with varying frequencies on TFM. The upper quartile was employed to confirm contacts expressing dominant H pattern (*d*H pattern). Bispectral analysis and transfer function modeling were employed to assess nonlinear properties and signal propagation, respectively. The performance of the *d*H pattern in evaluating the EZ was compared with other ictal biomarkers.

**Results:**

Regardless of seizure onset patterns, the H pattern commonly occurred during early or late seizure propagation among 57 patients (81.4%). It harbored within specific EEG segments characterized by fast activity and irregular polyspikes. The H pattern often appeared simultaneously across different brain regions at a consistent fundamental frequency, highlighting a crucial stage in seizure propagation characterized by inter-regional synchronization. The *d*H pattern demonstrated greater nonlinearity compared to the non-*d*H pattern, as evidenced by bispectral analysis. The waveforms associated with the *d*H pattern were more stereotyped and showed increased skewness and/or asymmetry. Notably, the complete removal of areas exhibiting the *d*H pattern, but not high epileptogenicity index (≥ 0.3) or seizure onset zone, was independently associated with seizure freedom after surgery.

**Conclusions:**

The H pattern provides unique insights into ictal neural dynamics. Additionally, it is a novel and alternative approach for measuring the EZ over an extended ictal time window.

**Supplementary Information:**

The online version contains supplementary material available at 10.1186/s12916-026-04665-7.

## Key points

1. The harmonic pattern (H pattern) is commonly present in focal epileptic seizures and can help to improve the accuracy of EZ localization over an extended time window.

2. The H pattern is a spectral signature of waveform skewness or asymmetry. The dominant H pattern reflects a stronger nonlinearity of ictal EEG signals.

3. The H pattern can appear simultaneously in different areas with a consistent fundamental frequency, indicating a key stage of inter-regional synchronization.

**Supplementary Information:**

The online version contains supplementary material available at 10.1186/s12916-026-04665-7.

## Background

Epilepsy is a chronic neurological disorder characterized by the tendency to have recurrent spontaneous seizures, which are associated with complex and dynamic patterns of neuronal synchrony and asynchrony. The accurate localization of the epileptogenic zone (EZ) is critical for achieving favorable surgical outcomes in drug-resistant focal epilepsy [1, 2]. Stereo-EEG (SEEG) is the most precise method for EZ identification. However, the ictal patterns observed on SEEG exhibit significant heterogeneity, suggesting a complex, highly dynamic, and individualized nature of the ictal network [3, 4]. Moreover, emerging evidence has shifted the understanding of the EZ from a focal source to a complex and hierarchical network [2, 5]. Seizure propagation is not a passive conduction but rather determined by inter-regional connectivity properties [5–7]. Specific EEG features during seizure propagation, such as phase-locked high-gamma activity, provide valuable localizing information [8]. Currently, the interpretation of ictal EEG patterns primarily relies on visual inspection by experienced clinical neurophysiologists. The limited success rate of surgery, with only 50–70% of patients achieving long-term seizure freedom, is still considered unsatisfactory [9]. The method for EZ localization requires further optimization.

**Fig. 1:**
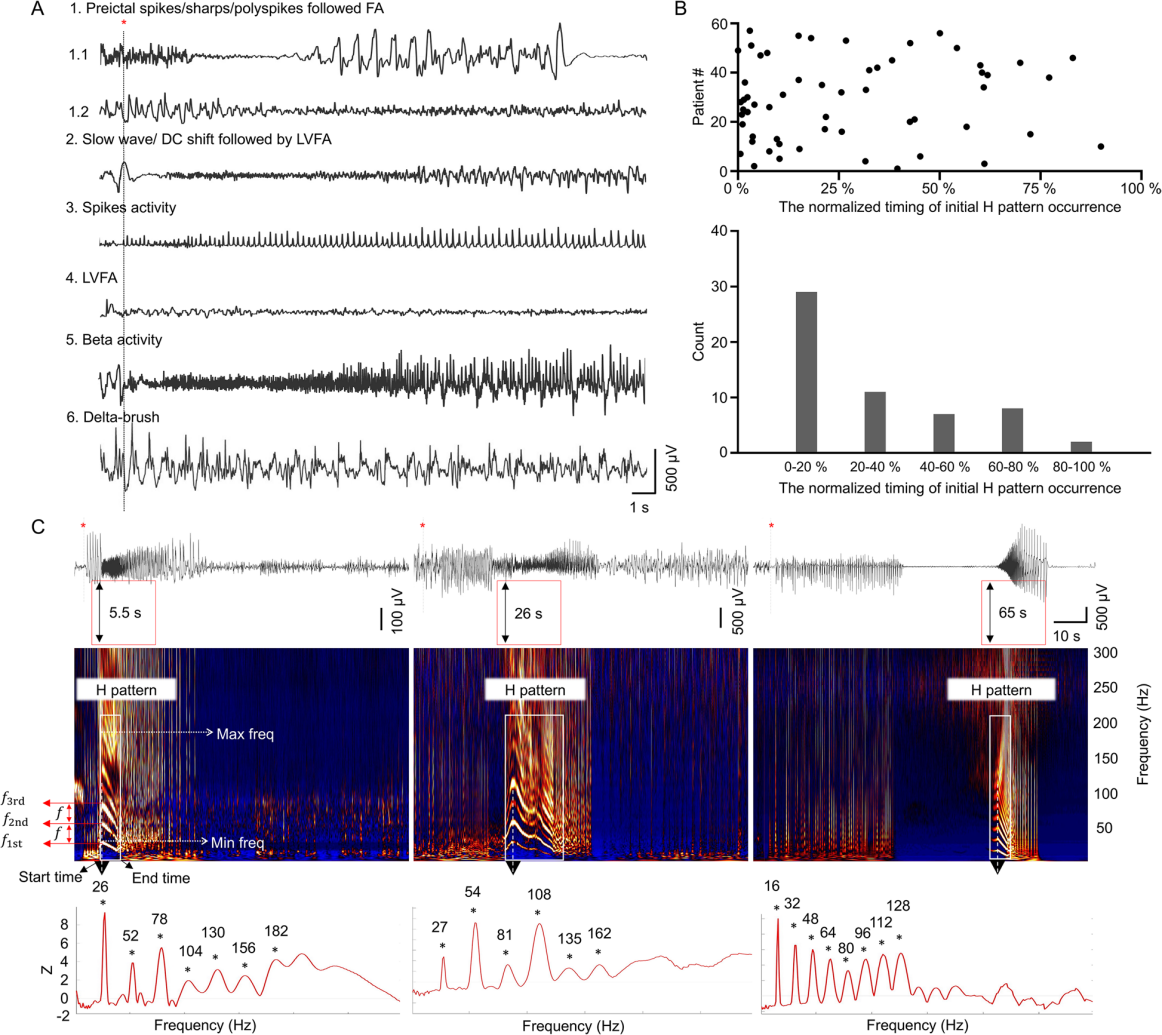
H patterns recorded in seizures with various ictal EEG onset patterns. **A** The six ictal onset patterns defined on SEEG. Pattern 1, preictal spikes/sharps/polyspikes following fast activity > 14 Hz (pattern 1.1: LVFA, amplitude < 30 μV; pattern 1.2: HVFA, amplitude > 30 μV); pattern 2, slow wave/DC shift followed by LVFA; pattern 3, spikes activity; pattern 4, LVFA; pattern 5, beta activity; pattern 6, delta-brush. **B** Distribution of the occurrence of the initial H pattern during seizures. Scatterplot showing the normalized timing of the initial H pattern start-point, expressed as a percentage of total seizure duration (top). Cumulative histogram showing the occurrence of the initial H pattern (bottom). **C** EEG (top) and corresponding TFM (middle) during seizures. The TFM shows the “harmonic pattern” (H pattern): multiple equidistant high-density, narrow bands with varying frequency over time. The initial H patterns presented at various seizure stages on TFM. The black double arrows in the red box mark the start time point of the H pattern. The power spectral density corresponding to the maximal frequency of the H pattern (the black arrow) demonstrates an equidistant distribution of the frequency bands (bottom). The frequency intervals were 26, 27, and 16 Hz, respectively. LVFA: low-voltage fast activity; HVFA: high-voltage fast activity; TFM: time–frequency map; Max freq: maximal frequency; Min freq: minimal frequency; *f*_1st_: the first frequency band; *f*_2nd_: the second frequency band; *f*_3rd_: the third frequency band; *f*: fundamental frequency (frequency interval). Red star: seizure onset

To improve EZ delineation, numerous quantitative EEG analysis methods have been developed, usually based on seizure onset stage [10]. Most approaches have focused on fast activity (FA, typically > 30 Hz), either as a standalone feature or in combination with other features [11–17]. For example, epileptogenicity index (EI) and epileptogenicity mapping measure the energy ratio changes between FA and slow wave activities during seizure onset, aiding in EZ localization [11, 12]. Emerging evidence highlights the importance of spectral architecture in ictal FA, not just its power [18, 19]. For instance, the ictal chirp, defined by Di Giacomo et al. as a high-power FA of at least 80 Hz that attenuates within 5 to 10 s, is a common pattern that may assist in EZ localization. However, its definition is not uniform across studies [18, 20, 21]. Grinenko et al. introduced EEG fingerprint as an ictal marker for EZ identification, noting that ictal FA consists of multiple narrow bands [19, 22]. Nevertheless, these methods demonstrate limited efficacy for slower seizure onset patterns, which account for 20–30% of cases [23–25], indicating the need for further investigation into ictal EEG spectral properties. Through comprehensive analysis of ictal SEEG spectral properties in focal epilepsy, we identified a unique spectral structure, referred to as the “harmonic pattern” (H pattern), featuring multiple, equidistant, high-density frequency bands that vary over time (Fig. 1). The H pattern is commonly observed in ictal EEG recordings from both experimental models of epilepsy and epileptic patients [20, 26, 27]. Notably, the presence of harmonics in EEG or local field potential (LFP) suggests nonlinear neural oscillations, which are relevant to neural communication and brain dynamics [28–30]. The H pattern may therefore provide straightforward insight into the nonlinear nature of the ictal networks. Here we systematically examined this phenomenon and evaluated its clinical significance in a cohort of patients with focal epilepsy undergoing SEEG evaluation. Furthermore, bispectral analysis was employed to interrogate the signal properties of the H pattern, thereby confirming its nonlinear nature.

## Methods

### SEEG evaluation

We conducted a review of consecutive patients with drug-resistant focal epilepsy who underwent SEEG evaluation followed by resective surgery at our epilepsy center between January 2014 and April 2021. This study was approved by the Medical Ethics Committee of our hospital (Study No. 2020–910), and written informed consent was obtained from all participants or their legal guardians. SEEG was performed using intracerebral multiple-contact electrodes (HKHS, Beijing, China), with contacts that were 2 mm in length, 0.8 mm in diameter, and 1.5 mm apart. SEEG signal was recorded by EEG systems with 256 channels (Nihon Kohden, Tokyo, Japan) or 128 channels (Xltek, Natus, Ontario, Canada) at a sampling rate of 1000 or 2000 Hz. Pre-implantation MRI and post-implantation CT were co-registered to locate each contact anatomically along each electrode trajectory.

SEEG electrodes were reviewed using a bipolar montage, which was constructed by subtracting signals from adjacent contacts along each electrode (e.g., 1–2, 2–3, 3–4; contact 1 is defined as the innermost (deepest), with contact numbers increasing to the outermost). Only contacts localized within the gray matter were included in the analysis. The seizure onset pattern (SOP) was assessed based on the earliest electrophysiological changes recorded from the electrode contacts. The seizure onset zone (SOZ) was defined as the cortical region exhibiting the earliest ictal activity, while the early propagation zone (PZ) comprised areas showing ictal spread within 3–5 s of seizure onset. All other brain regions were classified as other zones (OZ) [16, 31, 32]. Two independent clinical neurophysiologists assessed SOP, SOZ, PZ, and OZ. Any discrepancies were resolved through discussion with a third epileptologist to reach a consensus.

Postoperative evaluation involved co-registration of preoperative MRI and postoperative MRI scans to delineate resection boundaries. Electrode contacts within the resected area were classified as removed contacts. All patients were followed for at least 2 years after surgery, with surgical outcomes assessed using the Engel’s classification, classifying as Engel Ia (seizure-free, SF) or Engel Ib–IV (not seizure-free, NSF). Pathological classification was performed in accordance with the International League Against Epilepsy (ILAE) neuropathology guidelines [33–35].

### Quantitative EEG analysis

#### Identification of harmonic patterns

For each seizure, a 110-s SEEG epoch (− 10 s to + 100 s relative to seizure onset) was extracted. Time–frequency maps (TFM) were generated using the Morlet wavelet transform (center frequency *f*_0_ = 5–8 Hz, time resolution FWHM = 3 s), with a linear frequency resolution (1–300 Hz, 1 Hz step). Each TFM for each seizure was normalized using Z-score transformation, where power at each frequency was transformed by subtracting the mean and dividing by the standard deviation of the power over the entire 110-s window.

The H pattern was identified by its harmonic-like frequency distribution, consisting of multiple equidistant, high-density narrow bands that change over time (Fig. 1C). These bands, from low to high frequencies, are labeled sequentially (e.g., *f*_1st_, *f*_2nd_, *f*_3rd_). The consistent difference between adjacent bands defines the frequency interval, which is equal to the fundamental frequency (*f*), typically the lowest band’s frequency. The frequencies of these narrow bands align with integer multiples of *f*. The minimal and maximal frequencies are defined by the peak frequencies of the first and last identifiable bands, respectively, requiring clear separation from background activity in the TFM. Parameters of the H pattern, including minimal and maximal frequencies, as well as start and end times, were manually extracted using Brainstorm software. If multiple H patterns occurred within a single seizure, only the first one would be analyzed.

To further identify contacts exhibiting dominant H pattern, a threshold was set at the upper quartile (Q3, 75th percentile) of the total number of bands:

$$\mathrm{Q}3=\left(n+1\right)\times 3/4$$

where *n* is the highest band number in each seizure. Any contact with a band number of the H pattern exceeding this threshold would be designated as a “dominant H pattern” (*d*H pattern) contact; otherwise, it was labeled as a “non-dominant H pattern” (non-*d*H pattern) contact. Notably, the H patterns constitute intrinsic features of the signal, showing no dependency on the choice of time–frequency decomposition method or normalization procedures (Additional file 1: Fig. S1). The dominance of harmonic patterns is not determined by ictal discharge power, as substantial harmonics can occur with low discharge power or low EI (Additional file 1: Fig. S2).

#### Analysis of the signal properties of the H pattern

**Fig. 2:**
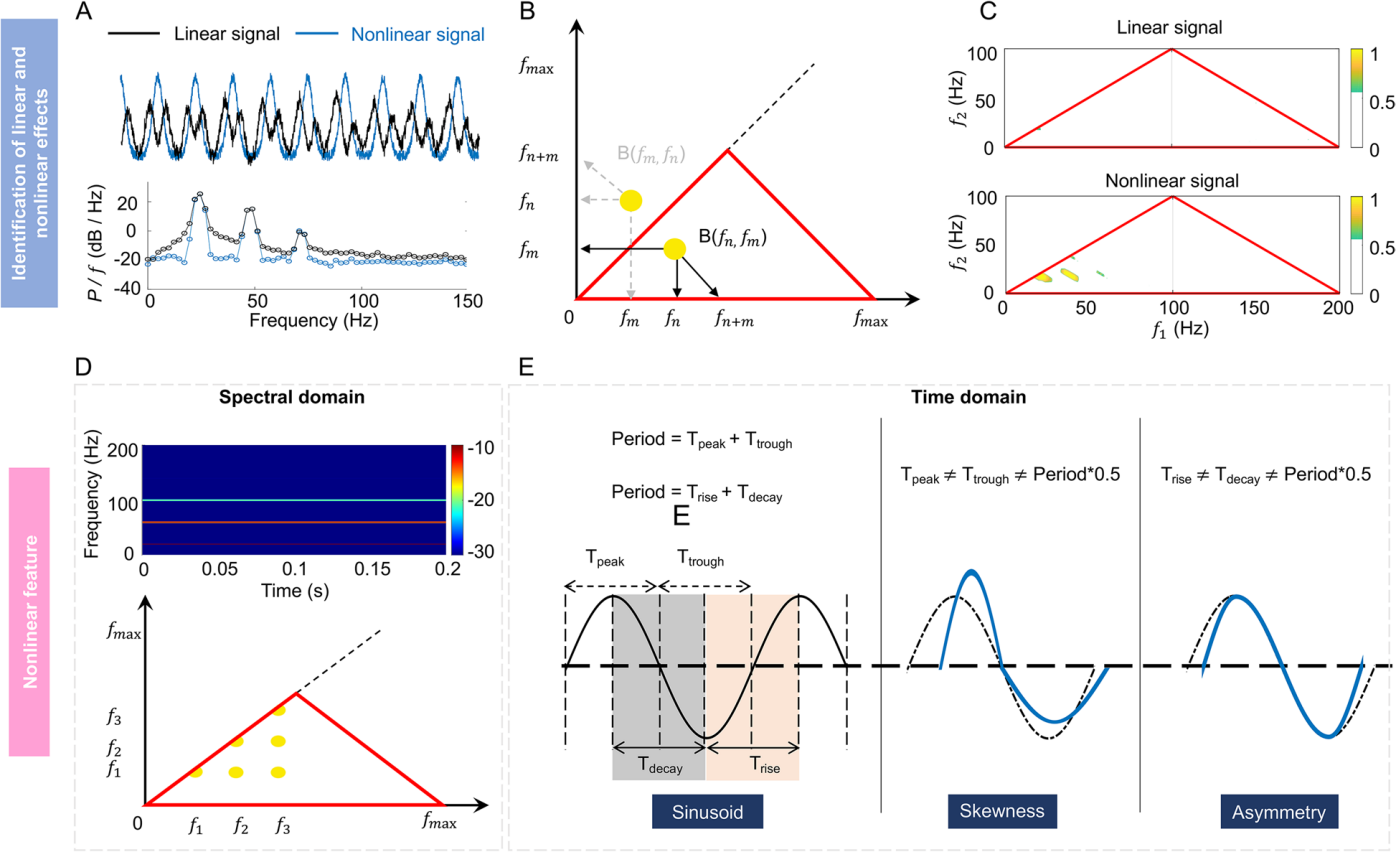
Analysis of the signal properties of the H pattern. Identification of linear and nonlinear effects: **A** linear and nonlinear signals (top) and their PSD (bottom). PSD analysis cannot distinguish between linear and nonlinear signals, as these oscillations have similar spectral density characteristics. **B** Bispectral symmetry. The main diagonal is the axis of symmetry, with identical data on both sides. The red triangle represents nonredundant bispectral information. The peak in the bispectral estimate (yellow dot) represents a phase-coupled triplet (${f}_{n}, {f}_{m}, {f}_{n+m}$). If a bispectral peak (yellow dot) is on the first diagonal, then $m=n$, meaning that frequency ${f}_{n}$ is phase correlated to its second harmonic ${f}_{2n}={2f}_{n}$. **C** Normalized bispectrum of linear and nonlinear signals. Bispectral analysis can distinguish linear and nonlinear characteristics of signals: the normalized bispectrum and its components for linear signal are approximately zero, whereas nonlinear signal exhibits four prominent peaks. Features of nonlinear effects: **D** in the spectral domain, nonlinearity is expressed as a series of harmonics that are phase-locked to the fundamental frequency, as evident in both TFM (top) and bispectral analysis (bottom). **E** In the time domain, nonlinearity is manifested as the asymmetry and skewness of the waveform. The left side uses a sinusoidal wave example to illustrate the period, peak, trough, rise, and decay. The distorted blue waveforms on the right, relative to the sinusoidal wave (black dashed line), exemplify skewness and asymmetry. PSD: power spectral density; DFT: discrete Fourier transform; TFM: time–frequency map

Harmonics can originate from either linear or nonlinear phenomena, each exhibiting their intrinsic characteristics (Fig. 2A). Linear systems allow for the superposition of solutions, resulting in distinct harmonics. Conversely, nonlinear systems involve complex amplitude interactions that lead to phase correlations undetectable by spectral density alone. These phase correlations can be assessed through bispectral analysis; the basic formula is as follows:

$${B}_{n,m}=B\left({f}_{n},{f}_{m}\right)=\mathrm{E}[{G}_{n}{G}_{m}{G}_{m+n}^{*}]$$

Here,${f}_{n}$ and ${f}_{m}$ are two frequencies, ${G}_{n}$ and ${G}_{m}$ are the corresponding complex Fourier coefficient using the discrete Fourier transform (DFT), and * denotes the complex conjugate. The bispectrum ${B}_{n,m}$ represents the phase correlation between Fourier modes with frequencies ${f}_{n}, {f}_{m}$ and ${f}_{n+m}={f}_{n}+{f}_{m}$.

To eliminate the distortion induced by the variance distribution, the bispectrum can be normalized as follows:

$${b}_{n,m}=\frac{{B}_{n,m}}{{\left(\mathrm{E}[{\left|{G}_{n}{G}_{m}\right|}^{2}]\mathrm{E}[{\left|{G}_{n+m}\right|}^{2}]\right)}^{1/2}}$$

whose squared modulus and phase are termed bicoherence and biphase, respectively [29]. The bispectrum (including its normalized form) exhibits characteristic symmetries in the (${f}_{n}, {f}_{m}$) plane (Fig. 2B). The main diagonal serves as a symmetry axis [36]. The red triangle highlights the nonredundant bispectral information, which is crucial for interpreting bispectral distributions. At any point (${f}_{n}, {f}_{m}$), the bispectrum ${b}_{n,m}$ represents the phase correlation between the Fourier modes with ${f}_{n}$, ${f}_{m}$, and ${f}_{n+m}$. While it is zero in linear systems with independent coefficients, nonlinear systems display peaks at phase-related triads, indicating two-wave coupling (Fig. 2C).

To thoroughly analyze the nonlinear characteristics of the H pattern, we simultaneously measured the skewness and asymmetry of the waveforms. In the spectral domain, nonlinearity is expressed as the development of a chain of harmonics statistically phase coupled to fundamental frequency. In the time domain, it is expressed as the development of skewness and asymmetry in the waveform (Fig. 2D, E). Skewness indicates horizontal asymmetry, where the duration of peaks or troughs is not half the period. Asymmetry indicates vertical asymmetry, with rise or decay times also not being half the period. Skewness and asymmetry can be extracted through bispectral analysis. The normalized bispectrum’s real and imaginary parts encapsulate the “frequency distribution” of these waveform distortions. We will refer to the real and imaginary parts of the normalized bispectrum as the distributions of skewness and asymmetry, defined as follows:

$${\xi }_{n,m}=\mathrm{Re}\{{b}_{n,m}\}; {A}_{n,m}=\mathrm{Im}\{{b}_{n,m}\}$$

Here, Re{z} and Im{z} denote the real and imaginary parts of the complex number z, respectively. The sign of $\xi$ and $A$ ($\pm$) indicates the direction of the skewness or asymmetry [29].

Furthermore, original bipolar SEEG waveforms underwent detailed analysis. Signals were first detrended using the “loess” smoothing method in MATLAB. The detrended SEEG trace was then segmented into positive and negative wave phases using the zero line as the boundary. An additional amplitude threshold was applied to exclude noise-induced extrema. For each identified segment, a sinusoidal fit was performed. From the fitted period, the trough duration (T_trough_) and peak duration (T_peak_) were derived; these represent the durations of the negative and positive phases, respectively. Subsequently, each peak-trough pair was used to segment the waveform into rising (from trough to peak) and decaying (from peak to trough) phases. A separate sinusoidal function was fitted to each of these phases to estimate their durations, yielding T_rise_ and T_decay_. Notably, waveform parameters were defined using the bipolar montage channels to measure asymmetry and skewness. Although this montage can invert signal polarity across channels (e.g., swapping troughs and peaks), the metrics of waveform asymmetry and skewness remain unaffected. For instance, a waveform manifesting as “sharper peaks” becomes “sharper troughs” after polarity inversion, yet its intrinsic asymmetry is preserved.

EEG signals were processed by de-meaning, linear detrending, and dividing into 50% overlapping segments of approximately 0.5-s windows (${2}^{10}$-point windows at a sampling rate ${f}_{s}=2000$ Hz), achieving a frequency resolution of approximately $\Delta f=2$ Hz. The total intervals were around 7 s, yielding approximately 28 degrees of freedom (DOF). For the normalization ${b}_{n,m}$, the probability density function was approximated using the noncentral ${\chi }^{2}$ distribution [36, 37]. A confidence level distinguishes linear from nonlinear stochastic processes. With DOF = 28, zero-mean bicoherence, significant in this context, is $\left|b\right|<\sqrt{\frac{6}{\mathrm{DOF}}}\approx 0.46$ for a 95% confidence level [37, 38]. All calculations were implemented in MATLAB, utilizing its DFT functions. Bispectral estimates were computed using code based on modified functions from the HOSA toolbox.

#### Bidirectional signal propagation assessment using FitPercent

To investigate the propagation relationship between the *d*H pattern and non-*d*H pattern signals, we employed a transfer function-based approach. For each signal pair (*a*, *b*), one signal is designated as the input and the other as the fitting target; the process is then repeated with the roles reversed to evaluate the opposite direction.

For each direction, the optimal transfer function is selected by maximizing the fitting accuracy, quantified by the optimal fitting percentage (FitPercent) metric, given as

$$\mathrm{FitPercent}=100\left(1-\frac{\Vert {y}_{\mathrm{measured}}-{y}_{\mathrm{model}}\Vert }{\Vert {y}_{\mathrm{measured}}-\overline{{y }_{\mathrm{measured}}}\Vert }\right)$$

where ${y}_{\mathrm{measured}}$ represent the target output data, $\overline{{y }_{\mathrm{measured}}}$ denotes its mean, and ${y}_{\mathrm{model}}$ is the simulated or predicted response of the model. $\Vert *\Vert$ indicates the 2-norm of a vector. FitPercent varies between − Infinity (bad fit) to 100 (perfect fit). A higher FitPercent indicates that the input signal contains sufficient information to reconstruct the target signal, thereby suggesting a likely propagation from the input to the target. This bidirectional analysis enabled us to assess potential propagation pathways between the *d*H and the non-*d*H pattern activities in a data-driven manner.

#### Comparative analysis of the dH pattern and other ictal EEG markers

We systematically assessed the diagnostic performance of the H pattern alongside established ictal EEG markers, including EI, SOZ, chirp, and EEG fingerprint. Chirp and EEG fingerprint were identified as previously described, and EI value was calculated using the AnyWave plugin [11, 18, 19]. For EI analysis, we included only patients with ictal FA at onset and at least one contact with high EI (≥ 0.3). Since chirp and EEG fingerprint can be present across multiple brain regions and lack established thresholds for measuring epileptogenicity level, they were not included in the subsequent predictive performance analysis. For three markers (EI, SOZ, and H pattern), the biomarker-defined EZ (bdEZ) were defined as follows: contacts with high EI, contacts defined as SOZ, and contacts with *d*H pattern.

We evaluated the predictive performance of each marker using two complementary analyses. For EZ predictability assessment, using resected contacts from the SF patients as the reference standard, we classified contacts into four categories: true positives (TP, resected bdEZ), false positives (FP, non-resected bdEZ), true negatives (TN, non-resected non-bdEZ), and false negatives (FN, resected non-bdEZ). Sensitivity (TP / (TP + FN)), specificity (TN / (TN + FP)), precision (TP / (TP + FP)), and Youden’s index (*J* = sensitivity + specificity − 1) were calculated for each marker. For outcome predictability analysis, patients were categorized based on the completeness of bdEZ resection (Yes/No) and surgical outcome (SF/NSF): TP (Yes, SF), FP (Yes, NSF), TN (No, NSF), and FN (No, SF). Receiver operating characteristic (ROC) curves were plotted, and the area under the curve (AUC) was calculated to assess the predictive performance for surgical outcomes. Additionally, we computed the resection ratio (number of resected bdEZ divided by total bdEZ) [31], where a value of 1 indicates complete removal and 0 represents no removal.

### Statistical analysis

SPSS 24.0 was used for statistical analysis. Continuous variables were tested for homogeneity of variance and normality. Normal variables were presented as mean ± SD; non-normal variables were reported as medians (Q1–Q3). Categorical variables were displayed as frequencies. Two-group comparisons used Student’s *t*-tests or Mann–Whitney *U* tests, and three-group analyses employed one-way ANOVAs or Kruskal–Wallis tests. Comparisons between two paired groups were performed using paired Student’s *t*-tests or Wilcoxon signed-rank tests. Pearson’s chi-square or Fisher’s exact tests were used for categorical variables. Multivariate logistic regression controlled for confounders in the *d*H pattern resection ratio comparisons. Significance was set at *P* < 0.05 (Bonferroni-adjusted for multiple testing).

## Results

### Prevalence of the H pattern in ictal SEEG

We retrospectively reviewed SEEG recordings from 80 patients with drug-resistant epilepsy (from January 2014 to April 2021). After excluding 3 patients with incomplete data and 7 with non-localizable SOZ, 70 patients were included in the final analysis (Additional file 1: Fig. S3). Of these, 57 (81.4%) patients presented with at least one H pattern, while the remaining 13 (18.6%) patients showed no discernible H pattern (Table 1). The presence of H pattern was independent of SOP or presence of ictal fast activity (Fig. 1A, Table 1), with no significant clinical differences between patients with versus without the H pattern. Notably, the presence of H pattern was correlated with chirp and EEG fingerprint, but not with high EI (Table 1). Within those with H pattern, 39 (68.4%) achieved seizure freedom. No differences in various features were observed between the SF and NSF groups, except for number of electrodes and pathological findings (FCD II versus gliosis/non-specific findings, Table 2).

**Table 1:** Clinical characteristics and surgical outcomes of the patients with and without H pattern

	H pattern (***n*** = 57)	Non-H pattern (***n*** = 13)	*P*
Age at onset, y	10.4 ± 8.5	10.6 ± 5.7	0.896^a^
Gender, M, *n* (%)	31 (54.4%)	9 (69.2%)	0.371^b^
Age at surgery, y	23.6 ± 12.3	22.6 ± 8.7	0.731^a^
Epilepsy duration, y	13.3 ± 11.2	12.0 ± 6.3	0.591^a^
SOP			0.095^b^
Preictal spikes/sharps/polyspikes following fast activity	31 (69.2%)	5 (38.5%)	
Slow wave/DC shift followed by LVFA	10 (17.5%)	1 (0.08%)	
Spikes activity	7 (12.3%)	5 (38.5%)	
LVFA	6 (10.5%)	1 (0.08%)	
Beta activity	3 (0.05%)	0 (0.0%)	
Delta-brush	0 (0.0%)	1 (0.08%)	
Epileptogenic lesion on MRI, *n* (%)	49 (85.9%)	10 (76.9%)	0.416^b^
Number of electrodes, *n*	8 (6–10)	10 (7–12)	0.086^c^
Number of contacts, *n*	96 (76–120)	108 (129.5–74.5)	0.287^c^
Follow up, *n*	88.8 (72.9–116)	83.9 (123.9–65.4)	0.71^c^
Localization, *n* (%)			0.475^b^
Temporal	20 (35.1%)	3 (23.1%)	
Frontal	24 (42.1%)	5 (38.5%)	
Parietal	4 (7.0%)	3 (23.1%)	
Occipital	4 (7.0%)	1 (0.08%)	
Multi-lobar	5 (8.8%)	1 (0.08%)	
Pathology, *n* (%)			0.342^b^
Hippocampal sclerosis	6 (10.5%)	0 (0.0%)	
Focal cortical dysplasia type II	28 (49.1%)	5 (38.5%)	
Focal cortical dysplasia (non-type II)	11 (19.3%)	3 (23.1%)	
Tumor	2 (3.5%)	2 (15.4%)	
Gliosis/non-specific	10 (17.5%)	3 (23.1%)	
High EI, *n* (%)	45 (78.9%)	9 (69.2%)	0.476^b^
Chirp, *n* (%)	46 (80.7%)	3 (23%)	0.000^b^
Fingerprint, *n* (%)	24 (42.1%)	0 (0.0%)	0.003^b^
Seizure-free, *n* (%)	39 ((68.4%)	9 (69.2%)	1.000^b^

Data are presented as the number of patients for categorized variables and the mean ± standard deviation for continuous variables

Abbreviations: *M* male, *y *years, *FA *fast activity, *PS *periodic spike, *L *left, *MRI *magnetic resonance imaging

^a^Unpaired two-tailed *t*-tests

^b^Fisher’s exact test

^c^Mann-Whitney *U* tests. Notably, subtle lesions on MRI also include epileptogenic lesions

**Table 2:** Clinical characteristics comparison of patients with H pattern by prognosis

	Total (***n*** = 57)	Seizure-free (Engel class Ia) (***n*** = 39)	Not seizure-free (> Engel class Ia) (***n*** = 18)	*P* value
Age at onset, y	10.4 ± 8.5	9.4 ± 7.5	12.6 ± 10.2	0.193^a^
Gender, M, *n* (%)	31 (54.4%)	20 (51.3%)	11 (61.1%)	0.489^b^
Age at surgery, y	23.6 ± 12.3	23.6 ± 12.8	23.7 ± 11.3	0.991^a^
Epilepsy duration, y	13.3 ± 11.2	14.2 ± 11.8	11.1 ± 9.6	0.329^a^
EEG segments with H pattern, FA/PS	30/27	20/19	10/8	0.764^b^
Lateralization, L, *n* (%)	26 (45.6%)	18 (46.2%)	8 (44.4%)	0.904^b^
Epileptogenic lesion on MRI, *n* (%)	49 (85.9%)	35 (89.7%)	14 (77.8%)	0.246^c^
Number of electrodes, *n*	8 (6–10)	8 (6–9)	9.5 (8–11.3)	0.001^d^
Number of contacts, *n*	96 (76–120)	90 (72–114)	110.5 (87.5–123.5)	0.09^d^
Follow up, m	88.8 (72.9–116)	85.0 (73.1–116.8)	88.9 (70.3–104.5)	0.757^d^
Localization, *n* (%)				0.243^c^
Temporal	20 (35.1%)	10 (25.6%)	10 (55.6%)	
Frontal	24 (42.1%)	18 (46.2%)	6 (33.3%)	
Parietal	4 (7.0%)	3 (7.7%)	1 (5.6%)	
Occipital	4 (7.0%)	4 (10.3%)	0 (0.0%)	
Multi-lobar	5 (8.8%)	4 (10.3%)	1 (5.6%)	
Pathology, *n* (%)				0.004^c^
Hippocampal sclerosis	6 (10.5%)	5 (12.8%)	1 (5.6%)	
Focal cortical dysplasia type II	28 (49.1%)	23 (59%)	5 (27.8%)	
Focal cortical dysplasia (non-type II)	11 (19.3%)	7 (17.9%)^e^	4 (22.2%)^e^	
Tumor	2 (3.5%)	2 (5.1%)	0 (0.0%)	
Gliosis/non-specific	10 (17.5%)	2 (5.1%)^e^	8 (44.4%)^f^	
High EI, *n* (%)	45 (78.9%)	33 (84.6%)	12 (66.7%)	0.165
Chirp, *n* (%)	46 (80.7%)	32 (82.1%)	14 (77.8%)	0.728
Fingerprint, *n* (%)	24 (42.1%)	17 (43.6%)	7 (38.9%)	0.781

Data are presented as the number of patients for categorized variables and the mean ± standard deviation for continuous variables

Abbreviations: *M *male, *y* years, *FA *fast activity, *PS *periodic spike, *L *left, *MRI *magnetic resonance imaging

^a^Unpaired two-tailed *t*-tests

^b^Pearson’s chi-square test

^c^Fisher’s exact test

^d^Mann-Whitney *U* tests

^e & f^Bonferroni correction. Notably, subtle lesions on MRI also include epileptogenic lesions

The initial H pattern emerged at 13.04 (2.8–24.2) s after seizure onset. Defined by seizure progression phases (early stage: 0–20%; late stage: 80–100%). The emergence of H patterns was not limited to the early stage and occasionally extended to the late stage (Fig. 1B, C). Two distinct H pattern subtypes were identified: (1) the FA-H pattern (30/57, 52.6%), embedded in fast activity (> 25 Hz), and (2) the PS-H pattern (27/57, 47.4%), within irregular polyspikes (> 5 Hz). Compared to the PS-H pattern, the FA-H pattern displayed fewer frequency bands, a higher frequency interval, earlier start and end times, and greater minimal and maximal frequencies (Additional file 1: Fig. S4). In terms of prognosis, localization, and pathological features, there were no significant differences between the FA-H and PS-H patterns. However, the correlation between the FA-H pattern and high EI was significantly stronger than that between PS-H pattern and high EI (*P* < 0.0001, 96.7% vs. 55.2%). Among all H patterns, the frequency bands demonstrated dynamic temporal evolution, primarily characterized by descending or bell-shaped trajectories, though rare ascending trends were also observed prior to their eventual dissipation. H patterns with downward or bell-shaped trajectories were highly concordant with chirp.

### Signal properties of the H pattern

**Fig. 3:**
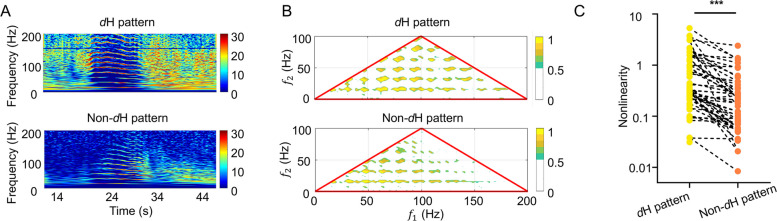
Bispectral analysis of the H patterns. **A** Example of the *d*H and non-*d*H patterns. **B** Example of normalized bispectrum for the *d*H and non-*d*H patterns. Compared to the non-*d*H pattern, the *d*H pattern exhibits more bicoherence peaks, signifying stronger harmonic phase coupling. **C** Comparison of bispectral values between the *d*H patterns and non-*d*H patterns in group data. The *d*H pattern showed stronger nonlinearity than the non-*d*H pattern (0.32 (0.21–1.15) vs. 0.12 (0.33–0.05), *P* < 0.0001, nonparametric Mann–Whitney)

To further investigate the factors contributing to the emergence of the H pattern, we analyzed the signal properties in both the frequency and time domains. In the frequency domain, bispectral analysis revealed that the emergence of the H pattern is due to nonlinear effects, with the *d*H pattern exhibited stronger nonlinear effects compared to the non-*d*H pattern (Fig. 3A–C).

Nonlinearity was also evident in the time domain through waveform distortions, characterized by skewness and asymmetry (Fig. 2D, E). In the following sections, we will carefully investigate the waveform characteristics of the EEG signals corresponding to the H pattern through four representative cases.

#### Stronger skewness underlying dH pattern

**Fig. 4:**
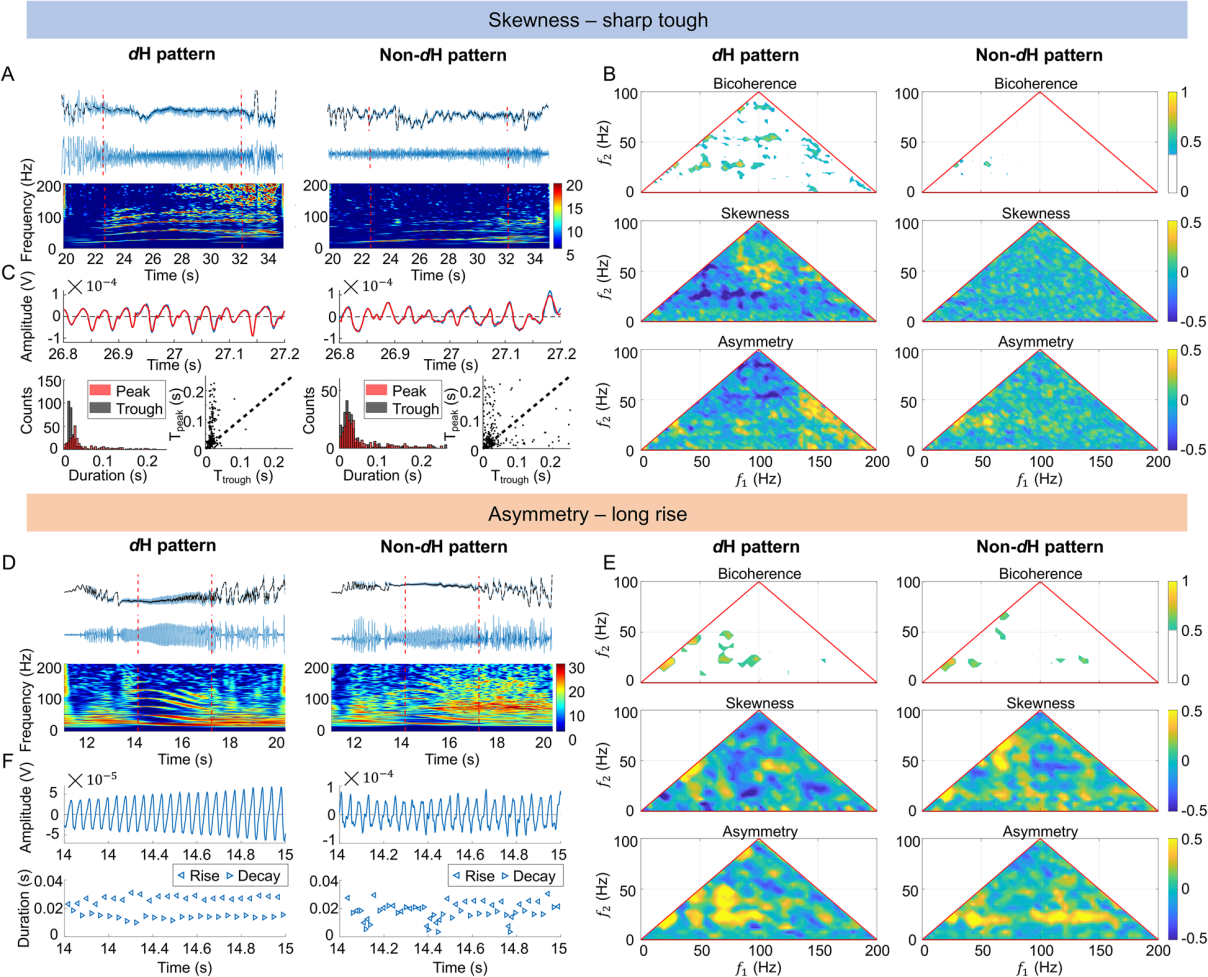
Waveform analysis of the H pattern. The *d*H pattern is attributed to stronger skewness (sharper trough): **A** Original SEEG signals in the *d*H/non-*d*H patterns (top), detrended SEEG signals (middle), and TFM (bottom); **B** bispectral analysis for the *d*H/non-*d*H patterns. **C** Comparison of peak and trough. Top: peaks and troughs (blue lines) in the *d*H/non-*d*H patterns are separately fitted by sin waves (red lines), respectively; bottom: histogram (left) and scatter plot (right) depicting the distribution of T_peak_ vs. T_trough_ for the *d*H/non-*d*H patterns. The *d*H pattern is attributed to stronger asymmetry (longer rise): **D** Original SEEG signals in the *d*H/non-*d*H patterns (top), detrended SEEG signals (middle), and TFM (bottom). **E** Bispectral analysis for the *d*H/non-*d*H patterns. **F** Comparison of rise and decay. Enlarged plots of detrended SEEG signals (top) and the corresponding rise (left triangles) and decay (right triangles) (bottom). The black dashed line in **A** and **D** represents the smoothed signals. The time with H pattern is marked by the red dotted vertical lines

In the first case involving the FA-H pattern, the *d*H pattern displayed more distinct bands compared to the non-*d*H pattern, which was attributed to waveform skewness (Fig. 4A–C). In the illustrated channel, the *d*H pattern consistently exhibited shorter T_trough_ than T_peak_, contributing to a more stereotyped waveform morphology (Fig. 4C). Bispectral analysis confirmed that the *d*H pattern had stronger bicoherence and skewness, while these features were less pronounced in the non-*d*H pattern (Fig. 4B). We constructed a simple waveform with sharper troughs, which exhibited pronounced harmonics. The corresponding bispectral analysis showed strong bicoherence and skewness, confirming that waveform skewness contributes to the emergence of the H pattern (Additional file 1: Fig. S5A–C, left).

#### Stronger asymmetry underlying the dH pattern

In the illustrated channel of the second FA-H pattern case, the *d*H pattern was characterized by asymmetric waveforms, with T_rise_ consistently longer than T_decay_ (Fig. 4D–F). This asymmetry contributed to a stereotyped EEG waveform. Bispectral analysis revealed stronger bicoherence and asymmetry in the *d*H pattern compared to the non-*d*H pattern (Fig. 4E). A designed waveform with longer T_rise_ than T_decay_ also exhibited strong harmonics and bispectral characteristics, validating the role of asymmetric waveforms in the emergence of the H pattern (Additional file 1: Fig. S5A–C, right).

#### Stronger skewness and asymmetry underlying the dH pattern

The waveform associated with the H pattern sometimes concurrently manifested skewness and asymmetry. In one more case with an FA-H pattern, the *d*H pattern in the illustrated channel was characterized by sharp troughs and asymmetry (short rise, long decay) of waveforms (Additional file 1: Fig. S6A, C, D). The distorted waveforms consistently appeared for the *d*H pattern, causing a stereotyped morphology of the EEG segment. Bispectral analysis confirmed stronger bicoherence, skewness, and asymmetry in the *d*H pattern compared to the non-*d*H pattern (Additional file 1: Fig. S6B). Similar findings were observed in the PS-H pattern (Additional file 1: Results S1, Fig. S7).

In summary, bispectral analysis revealed the existence of oscillatory interactions in the H pattern, suggesting a nonlinear effect. In the time domain, the H pattern was the spectral signature of the shape change of EEG waveforms.

### Spatial properties of the H pattern

The H pattern can be distributed across multiple brain regions. To investigate its spatial properties, we analyzed the distribution features of the H pattern in the SOZ, PZ, and OZ, and examined the directionality of its signal propagation.

#### Zonal distribution

**Fig. 5:**
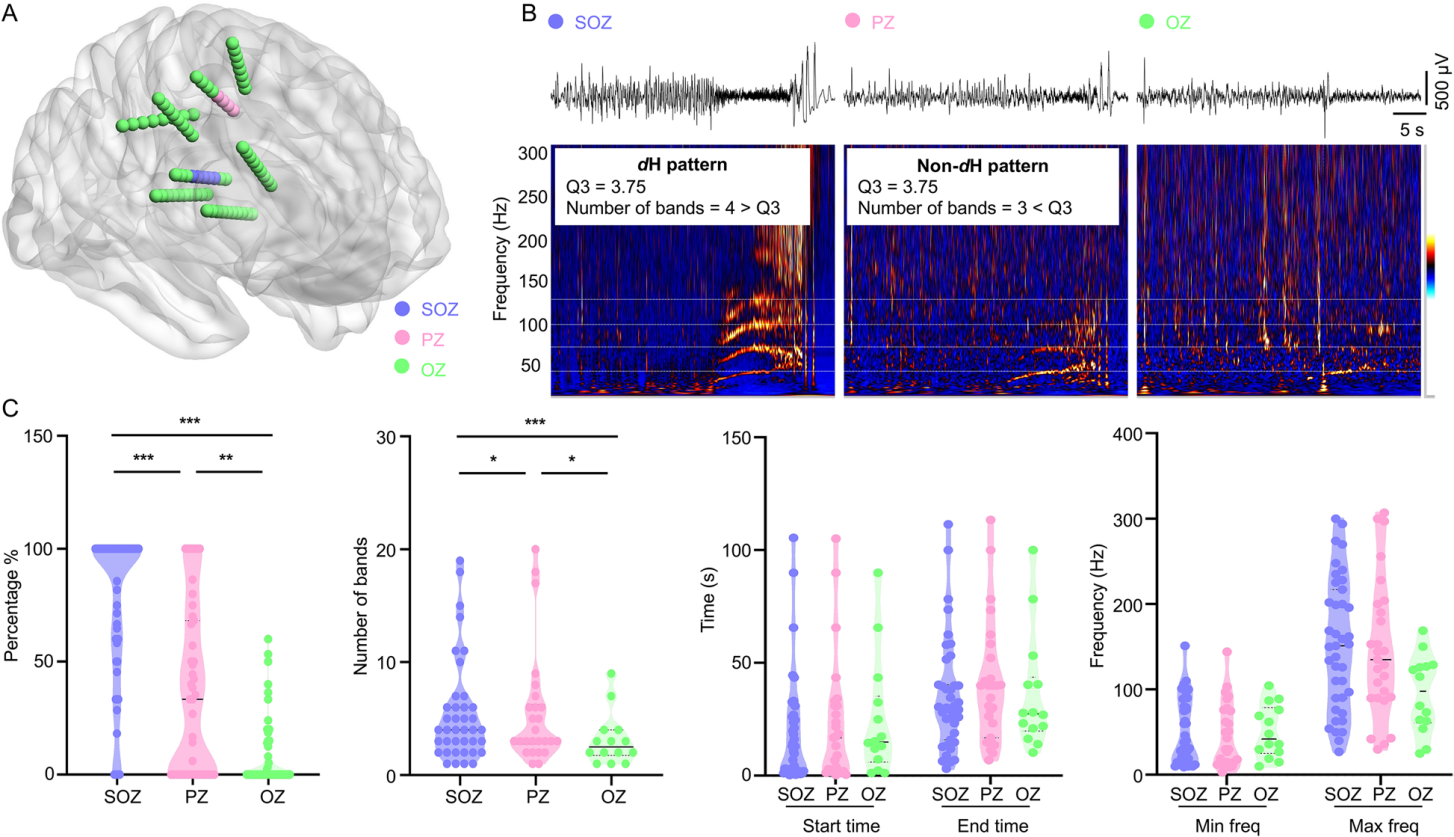
Distribution of the H pattern. **A** Anatomical distribution of SOZ, PZ, and OZ in a patient (left panel). In this case, SOZ is located in the precentral gyrus with early propagation to the postcentral gyrus. The right panel (**B**) shows the corresponding EEG recordings from SOZ, PZ, and OZ, along with the observed H pattern. **C** Comparison of parameters among SOZ, PZ, and OZ in group data (patient level). The distribution percentage and the number of bands of H pattern decrease in the order of SOZ, PZ, and OZ (percentage %: SOZ: 100 (60–100), PZ: 33 (0–68), OZ: 0 (0–13), SOZ vs. PZ, *P* < 0.001; SOZ vs. OZ, *P* < 0.001; PZ vs. OZ, *P* = 0.004. Number of bands: SOZ: 4 (2–6), PZ: 2 (0–4), OZ: 0 (0–2), *P* = 0.014; SOZ vs. OZ, *P* < 0.001; PZ vs. OZ, *P* = 0.012, nonparametric Kruskal–Wallis tests, after Bonferroni correction). *P < 0.05; **P < 0.01; ***P < 0.001. Max freq: maximal frequency; Min freq: minimal frequency; SOZ: seizure onset zone; PZ: early propagation zone; OZ: other zone; *d*H pattern: dominant H pattern

The proportion of contacts displaying the H pattern decreased from SOZ to PZ and then to OZ. Likewise, the number of bands exhibited a similar decreasing trend in each respective zone (Fig. 5C). The H pattern commonly occurred in the SOZ, PZ, and sometimes in OZ at very close time points, displaying nearly identical frequency intervals. The H pattern’s end time and minimal/maximal frequencies did not vary across regions (Fig. 5).

#### Signal propagation analysis

**Fig. 6:**
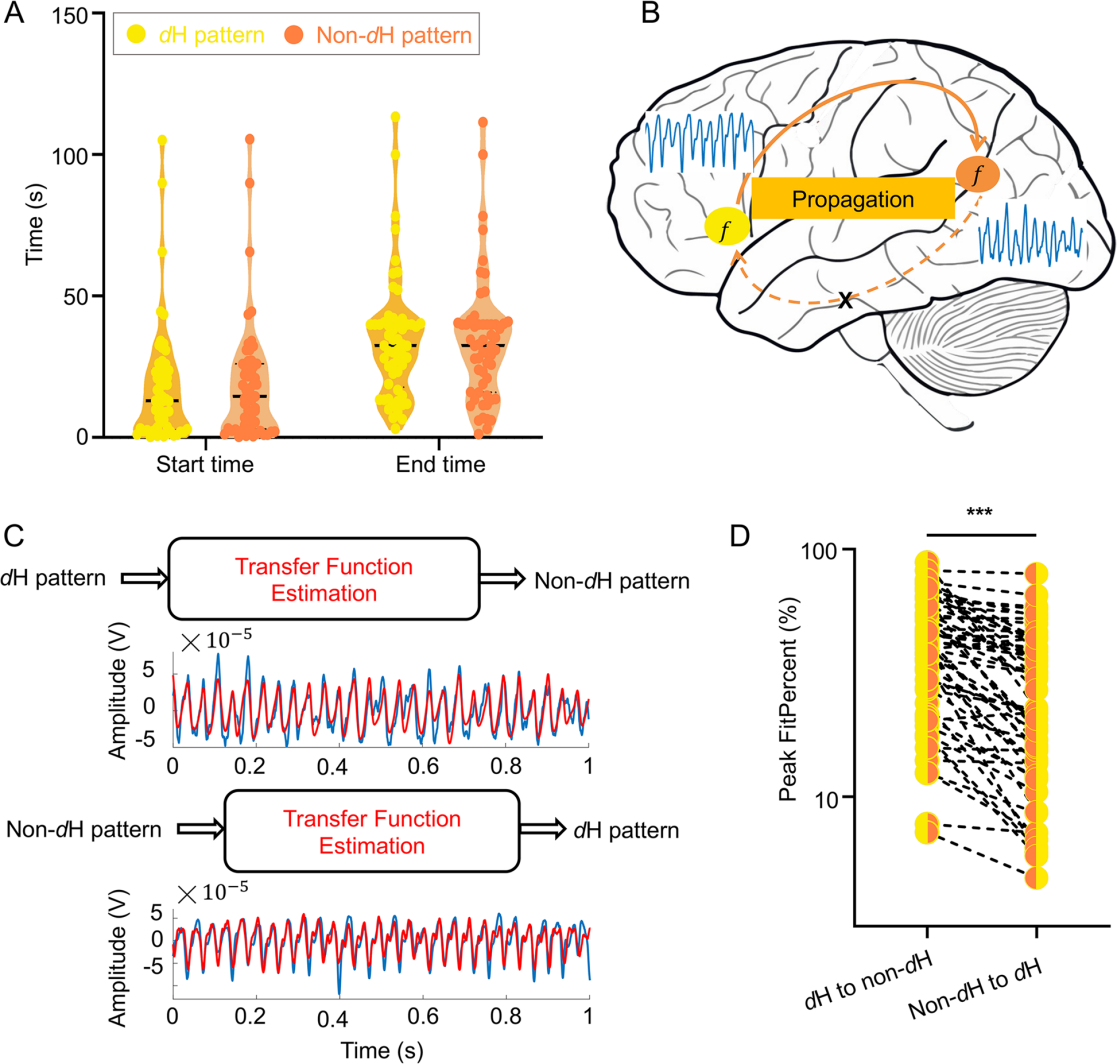
Signal propagation analysis of the H pattern. **A** Comparison of start and end times between the *d*H pattern and the non-*d*H pattern. **B** Schematic of signal propagation from region with the* d*H pattern to one with the non-*d*H pattern but not the opposite. The two regions share the same *f*. **C** Top: estimating a transfer function with the input of *d*H pattern to fit non-*d*H pattern. Bottom: estimating a transfer function with the input of non-*d*H pattern to fit *d*H pattern (blue: the input signal, red: the fitting signal). **D** Comparison of peak FitPercent between propagation from the *d*H pattern to the non-*d*H pattern and its opposite. The *d*H to non-*d*H direction showed a higher peak FitPercent (42.44 (24.69–59.13) vs. 22.68 (13.74–42.46) %, *P* < 0.0001, Wilcoxon signed-rank). *P < 0.05; **P < 0.01; ***P < 0.001. *f*: fundamental frequency

To further investigate the signal propagation properties of the H pattern, we classified it into the *d*H and non-*d*H patterns. Both *d*H and non-*d*H patterns typically share the same *f*, indicating synchronized oscillations between these regions and suggesting an underlying connection. This synchronization likely results from signal propagation between these regions. To test this hypothesis, we initially compared the start and end times of both patterns but no statistically significant differences were found (Fig. 6A). This may be attributed to the limited temporal resolution of the current measurements, which is insufficient to precisely resolve such rapid propagation processes. Consequently, we proceeded to validate our hypothesis through waveform similarity analysis. We used transfer functions to evaluate the relationship between the *d*H and non-*d*H patterns. The results revealed that the peak FitPercent was higher when estimating propagation from the *d*H pattern to the non-*d*H pattern, compared to the reverse direction (Fig. 6). This suggests that the *d*H pattern is more likely to propagate to the non-*d*H pattern, while propagation in the opposite direction occurs less frequently. This finding also implies that the *d*H pattern contains more information than the non-*d*H pattern.

### Potential values of the *d*H pattern in EZ localization

**Fig. 7:**
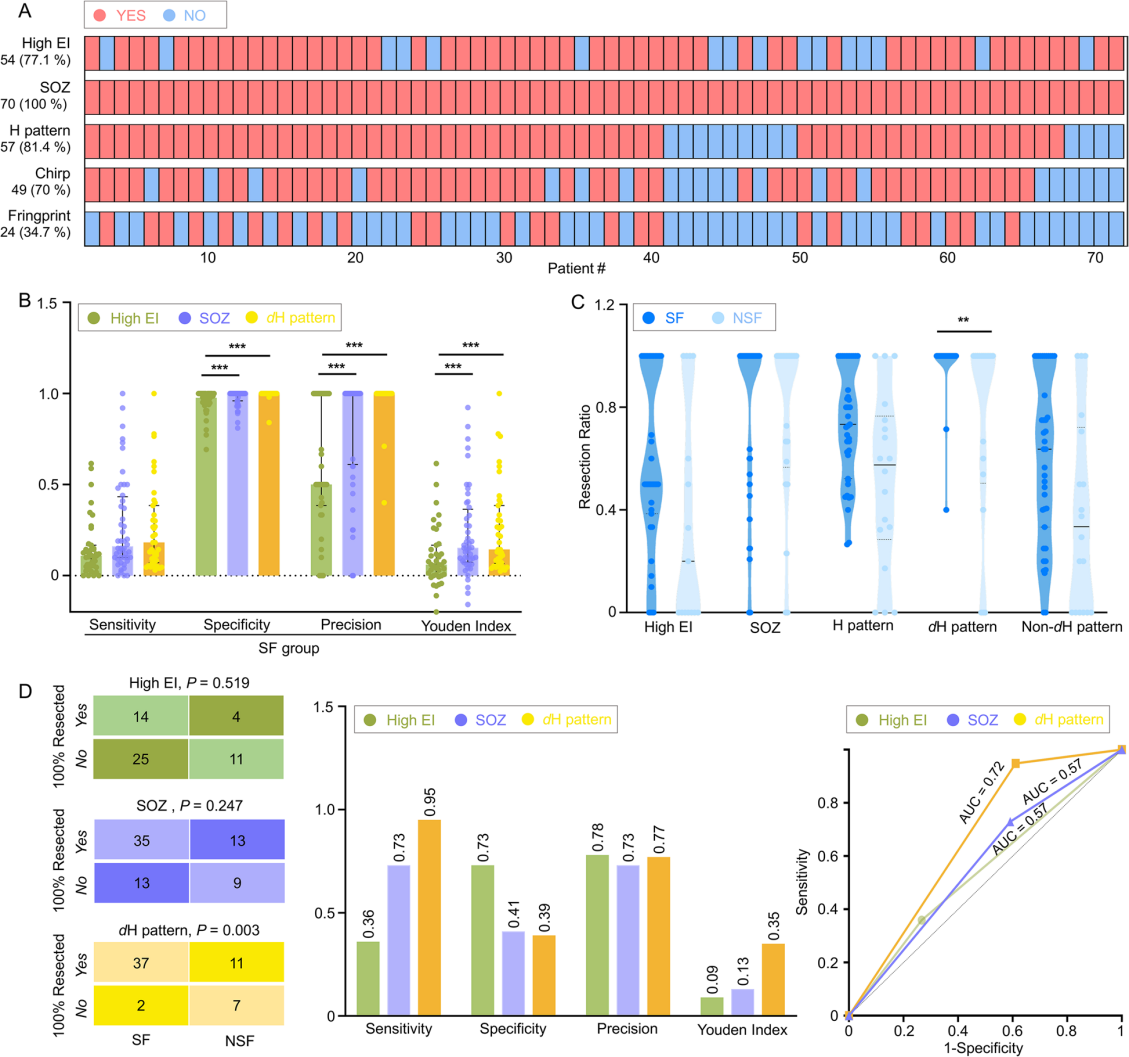
The localization value of the *d*H pattern. **A** The presence of five ictal markers in the 70 patients. Red = presence; blue = absence. For high EI, presence was defined by at least one contact with high EI; absence was defined by no high EI contacts or absence of FA at onset. **B** Statistics in predicting EZ in the SF group. Compared with high EI, the *d*H pattern and SOZ demonstrated higher specificity, precision, and Youden index (specificity: high EI: 0.98 (0.95–1), SOZ: 1.00 (0.96–1), *d*H pattern: 1 (1–1), high EI vs. SOZ, *P* = 0.005; high EI vs. *d*H pattern, *P* < 0.001; SOZ vs. *d*H pattern, *P* = 0.057. Precision: high EI: 0.50 (0.38–1), SOZ: 1.00 (0.6–1), *d*H pattern: 1.0 (1–1), high EI vs. SOZ, *P* = 0.001; high EI vs. *d*H pattern, *P* < 0.001; SOZ vs. *d*H pattern, *P* = 0.074. Youden index: high EI: 0.06 (0–0.17), SOZ: 0.15 (0.08–0.37), *d*H pattern: 0.14 (0.39–0.07), high EI vs. SOZ, *P* = 0.006; high EI vs. *d*H pattern, *P* < 0.001; SOZ vs. *d*H pattern, *P* = 0.719. Nonparametric Kruskal–Wallis tests, after Bonferroni correction). **C** Resection ratio of the ictal markers in SF and NSF group. Only the resection ratio of the *d*H pattern differed significantly between the two groups (1.00 (1–1) vs. 1.00 (0.5–1), *P* = 0.005, nonparametric Mann–Whitney *U* test, after Bonferroni correction). **D** Statistics of ictal quantitative markers in predicting outcome. The *d*H pattern demonstrated superior predictive performance for seizure freedom, with the highest sensitivity (0.95), Youden index (0.35), and AUC (0.72; 95% CI: 0.57–0.84; *P* = 0.026) among three markers. *P < 0.05; **P < 0.01; ***P < 0.001

All 70 patients exhibited an identifiable SOZ. Most patients demonstrated the H pattern (81.4%), high EI (77.1%), and chirp (70%), while fewer exhibited the EEG fingerprint (34.3%) (Fig. 7A). In terms of quantitative analysis, the overlap of bdEZ was used to assess concordance. Pairwise bdEZ overlap analysis revealed partial concordance between high EI and SOZ (31/54, 57.4%), *d*H pattern and SOZ (28/57, 49.1%), and high EI and *d*H pattern (21/45, 46.7%). Triple-marker analysis demonstrated partial overlap in 82.2% (37/45) of patients (Additional file 1: Fig. S8). No statistical difference was found in the number of bdEZ contacts among the three markers (Additional file 1: Table S1).

In predicting the EZ among SF patients, high EI demonstrated inferior specificity, precision, and Youden’s index compared to SOZ and *d*H pattern (Fig. 7B). For outcome prediction, the *d*H pattern exhibited superior sensitivity of 0.95 and Youden’s index of 0.35. ROC analysis revealed that the *d*H pattern had the highest AUC (0.72; 95% CI: 0.57–0.84; *P* = 0.026), whereas the other two markers had lower and non-significant AUCs of 0.57 (both *P* > 0.05). However, no significant differences were found among the three AUCs according to the DeLong test (Fig. 7D).

Furthermore, no difference in resection ratio was found except for *d*H pattern in the SF group compared to the NSF group (Fig. 7C). On multivariate analysis, after adjusting for potential confounders of pathology and number of electrodes, a complete resection of areas expressing the *d*H pattern was significantly associated with a higher chance of seizure freedom (OR = 32.33; 95% CI: 2.29–455.88; *P* = 0.01).

## Discussion

**Fig. 8:**
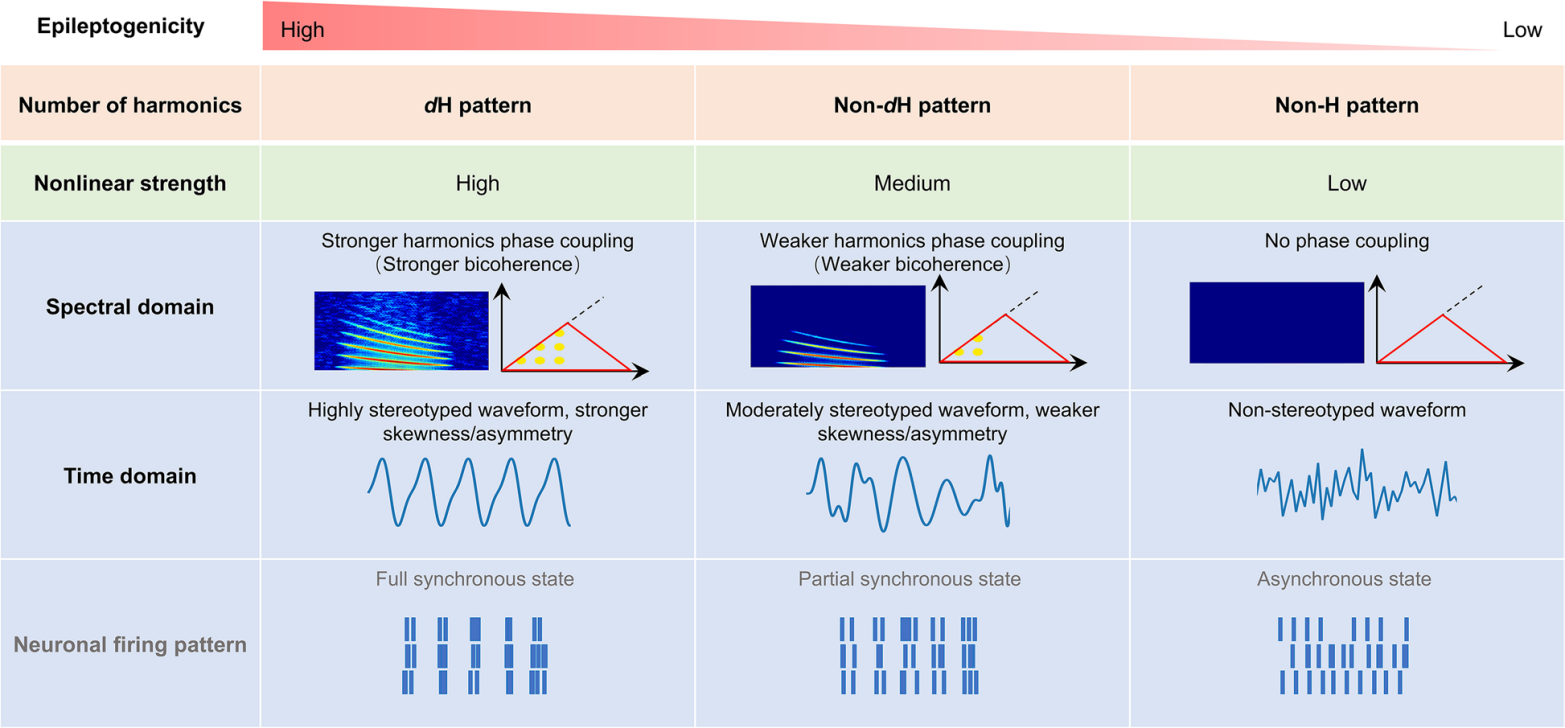
Summary of the nonlinear features for H pattern. The epileptogenicity, as reflected by the nonlinear features of H pattern, exhibits a clear gradient of decrease from the *d*H pattern to non-*d*H pattern and is notably diminished in the non-H pattern. In the spectral domain, this is seen as a decline in harmonic numbers in TFM and coupled peaks in bispectral analysis. In the time domain, the decline is marked by reduced skewness and/or asymmetry of waveforms, with the *d*H pattern correlated with more stereotyped waveforms. It is hypothesized that the harmonic nonlinearity reflects the synchronization level of neuronal firing, which decreases from the* d*H pattern to non-*d*H pattern and is absent in the non-H pattern

As a distinct spectral feature of ictal SEEG signals, the H pattern was commonly observed in most patients with various SOPs, whether early or late in seizure propagation. Its analysis not only provides crucial insights into the dynamics of ictal neural activity but also offers valuable information about the EZ. Our important findings include: (1) the dominance of the H pattern is a promising feature that may aid in EZ localization, serving as a complementary tool to conventional evaluations; (2) the H patterns represent spectral signature of waveform skewness and/or asymmetry, with the *d*H pattern reflecting a stronger nonlinearity of ictal oscillations, which correlates with higher epileptogenicity (Fig. 8); (3) the H patterns can occur simultaneously in different cortical regions with consistent fundamental frequencies, suggesting they represent a pivotal stage of inter-regional synchronization during seizure propagation.

The H pattern represents a potential novel EEG biomarker of EZ. Its performance of ictal H pattern was non-inferior or better compared with that of high EI or SOZ in terms of EZ prediction and prognostic analysis. The resection ratio of the *d*H pattern was significantly higher in the SF group than NSF group. However, direct comparisons of AUC among the three quantitative markers revealed no significant differences, indicating that the predictive power of a single marker is limited. The presence of the H pattern did not change the overall surgical outcome; however, it may help EZ localization in the absence of other ictal markers. Integrating multiple ictal markers, together with interictal findings, is pertinent to improve EZ localization. Furthermore, our study revealed a high concordance between the H pattern and chirp. The definition of a chirp has evolved over time [18, 20, 21]. Initially described as “a brief signal with rapidly changing frequency content” on the TFM [20], the H pattern can—on this basis—be considered a specific type of chirp characterized by harmonic frequency components. This finding not only deepens our understanding of chirp phenomena but also provides a new analytical method for quantifying their epileptogenicity.

The FA-H and PS-H patterns manifested during different seizure stages. Compelling evidence indicates that the presence of fast activity is supported by a significant activation of inhibitory interneurons, combined with a transient shutdown of excitatory cells [39–44]. Therefore, the FA-H pattern may be predominantly supported by inhibitory interneurons. Irregular spiking usually occurs at the later stage of seizures and is linked to a decrease in inhibitory interneuron firing and an increase in excitatory neuron interactions [45]. The PS-H pattern may be supported by a more substantial involvement of excitatory neuron firing.

Harmonic phenomena are prevalent in EEG/LFP signals, potentially linked to specific brain functional states [46–48]. For instance, during non-rapid eye movement sleep, sleep spindles exhibit harmonic phenomena, with parameters changing in accordance with sleep stages, reflecting functional alterations in the thalamocortical reverberant network [49]. Sheremet et al. studied the LFP recorded in the rat hippocampus and found harmonics of theta activity, together with the corresponding waveforms, changed with the movement speed. But harmonic phenomena have not received adequate attention in the field of epilepsy [20, 26, 27]. Our findings reveal that harmonic features contain localizing information about the neural dynamics of seizures, enriching our understanding of the ictal network and underscoring the importance of considering its nonlinear aspects.

Neural oscillations are vital for understanding both normal and pathological brain states [50, 51]. However, traditional linear methods may miss the complexity of brain activity [30]. Increasing evidence suggests that neural oscillations often have nonlinear properties [29, 30]. Our study shows that ictal signal nonlinearity, indicated by bispectral analysis and waveform skewness/asymmetry, is quantifiable, with the *d*H pattern exhibiting greater nonlinearity. Some studies showed nonlinear analysis of ictal EEG using sophisticated algorithms could provide seizure localization information [16, 52]. However, the nonlinear feature of the H pattern can be directly visualized in both the time and spectral domains, offering significant practical application value. In addition, our findings highlight the importance of waveform analysis, which can complement linear methods of EZ localization based on EEG frequency and power. Notably, harmonics can originate from either linear or nonlinear phenomena. Although bispectral and waveform analyses confirmed the existence of nonlinear property of the H pattern, the potential role of linear property cannot be entirely ruled out. Future research is needed to further elucidate this aspect.

Many studies suggest that EEG/LFP waveforms can reflect the degree of correlated activity within neuronal populations. Sharp transients observed in spike-wave discharges are indicative of synchronous neuronal firing [53]. Recent research has shown that increased neuronal synchronization results in highly asymmetric waveforms, while reduced synchronization leads to more sinusoidal shapes [54]. Additionally, a stronger nonlinear component in the waveform often indicates greater neuronal synchronization in the cerebral cortex [55]. Our results show that the *d*H pattern exhibits greater skewness and/or asymmetry in its waveforms, with a more stereotyped waveform. This consistency suggests the repeated occurrence of the same oscillatory pattern. These findings imply that the H pattern may serve as an EEG signature of rhythmic and synchronized neuronal firing within local epileptic tissues (Fig. 8). Further research is imperative to elucidate the cellular and dynamic processes responsible for the diverse waveforms associated with the H pattern.

The H pattern represents a distinctive stage in seizure propagation and contains valuable information for EZ localization. Historically, seizure propagation was considered secondary, evidenced by delayed EEG discharges occurring outside the EZ. However, in reality, seizure propagation exhibits significant spatial and temporal variability, even within the same patient. The understanding of the propagation has been evolving. Certain EEG features during seizure evolution, such as phase-locked high-gamma activity, have been proven valuable for EZ localization [8]. Our data suggest that seizure propagation can also be reflected in the spectral properties of EEG. Specifically, the H pattern visually represents a unique stage of synchronization across brain regions, as evidenced by the consistent fundamental frequency observed across these regions. Whether it is *d*H or non-*d*H, the H pattern manifests simultaneously in spatially distributed areas. FitPercent analysis indicates that signals from regions exhibiting the *d*H pattern are more likely to propagate to regions with non-*d*H pattern, aligning with the gradient of epileptogenicity. Therefore, the network properties of the H pattern may be useful for exploring epileptogenic networks. Notably, the localizing accuracy of the *d*H pattern alone was comparable to conventional SOZ analysis. Integrating the propagation-related features with seizure onset activity could therefore yield complementary information, enabling a more comprehensive delineation of the epileptogenic network and improved localization precision.

The H pattern can be observed throughout the entire seizure phase and is closely associated with the rhythmic firing and dynamic synchronization of neurons in epileptic tissues. This finding is consistent with prior research indicating that neuronal synchronization during seizures is a dynamic process, characterized by alternating phases of synchronization and desynchronization, reaching its peak at seizure termination [56–58]. The sequential occurrence of multiple H patterns during some seizures likely reflects the dynamic fluctuations in synchronization, suggesting a dynamic change in synchronization throughout the seizure which required further investigation.

The study has several limitations. First, due to the heterogeneous morphology of H patterns across subjects and the current lack of automated identification, their visual recognition was inherently subjective to personal experience. Second, simultaneous unit recording was not performed, which limits the exploration of the cellular mechanisms underlying the H pattern. Moreover, multiple H patterns sometimes occur in the same seizure, but only the first occurrence was analyzed in this study. Further investigation is warranted for cases where H patterns appear consecutively. Third, using a threshold (Q3) to define the dominant H pattern may not accurately capture individual variability. Future studies could benefit from adopting a patient-specific approach to defining the dominant H pattern. Fourth, the clinical value of the H pattern as a potential biomarker requires further investigation, including its application across different types of epilepsy, the synergistic effect of combination with other biomarkers, and the differences in prognostic prediction capabilities between the H pattern subtypes. Lastly, we have not yet identified the specific factors associated with the absence of the H pattern. This suggests that the neurophysiological manifestations are highly heterogeneous across individuals. Further investigation into this issue is warranted. Additionally, spatial sampling remains an intrinsic limitation of any SEEG study.

## Conclusions

Our study defines a common and distinctive ictal spectral feature, termed the H pattern. Its dominance signifies specific waveforms and highlights the nonlinearity of ictal EEG signals. The H pattern imparts unique information about ictal seizure propagation as well as offers novel insights into EZ localization. Our data also provides evidence supporting an elongated time window for measuring EZ using quantitative EEG.

## Supplementary Information

Additional file 1: Results S1; Figures S1–S8; Table S1. Results S1 Stronger skewness and asymmetry underlying the *d*H pattern. Fig. S1 Illustration of non-normalized and normalized TFM using the Morlet wavelet transform and the multitaper method. Fig. S2 Representative illustration of the relationship between ictal discharge power and harmonic patterns. Fig. S3 Flow chart showing the inclusion and exclusion process of patients in our study. Fig. S4 Two types of EEG segments harboring H pattern. Fig. S5 Two simulated waves to validate harmonic components induced by waveform distortion. Fig. S6 The *d*H pattern attributed to stronger skewness and asymmetry of the FA waves. Fig. S7 The *d*H pattern attributed to stronger skewness and asymmetry of the PS waves. Fig. S8 Concordance of high EI, SOZ, and *d*H pattern. Table S1 Comparison of contact numbers for the three markers.

Additional file 2.

Additional file 3.

Additional file 4.

## Data Availability

The collected EEG data is used for seizure localization and to inform clinical decisions. The de-identified dataset supporting the study findings is available upon request from the corresponding author. For inquiries or to request the source data for the figures, please contact S.W. via email. EEG data analyses were performed using the freely available toolbox Brainstorm in combination with custom Matlab scripts, which are available at: https://github.com/chenx-epi/H-pattern.

## Abbreviations

EZ: Epileptogenic zone
SEEG: Stereo-EEG
FA: Fast activity
EI: Epileptogenicity index
H pattern: Harmonic pattern
LFP: Local field potential
SOP: Seizure onset pattern
SOZ: Seizure onset zone
PZ: Early propagation zone
OZ: Other zones
SF: Seizure-free
NSF: Not seizure-free
ILAE: International League Against Epilepsy
TFM: Time-frequency maps
*f*: Fundamental frequency
Q3: The upper quartile
*d*H pattern: Dominant H pattern
non-*d*H pattern: Non-dominant H pattern
DFT: Discrete Fourier transform
DOF: Degrees of freedom
FitPercent: Fitting percentage
bdEZ: Biomarker-defined EZ
TP: True positives
FP: False positives
TN: True negatives
FN: False negatives
ROC: Receiver operating characteristic
LVFA: Low-voltage fast activity
HVFA: High-voltage fast activity
Max freq: Maximal frequency
Min freq: Minimal frequency
PSD: Power spectral density
PS: Periodic spike
