# Supplementary material for: Harmonic patterns embedded in ictal EEG signals in focal epilepsy: new insight into the epileptogenic zone
Source: BMC Med. 2026 Jan 28;24:115. doi: 10.1186/s12916-026-04665-7 (PMC12924499; doi:10.1186/s12916-026-04665-7)
Supplement: Supplementary file 3 — Additional file 3. [file 12916_2026_4665_MOESM3_ESM.docx]

Supplementary Materials for

**Harmonic patterns embedded in ictal EEG signals in focal epilepsy: new insight into the epileptogenic zone**

Lingli Hu, *et al.*

*Corresponding author: Shuang Wang. E-mail: [wangs77@zju.edu.cn](mailto:wangs77@zju.edu.cn).

Dongping Yang. E-mail: dpyang@zhejianglab.com.

**Supplementary Text**

**Supplementary Results**

**Stronger skewness and asymmetry underlying the *d*H pattern**

We presented a case with the PS-H pattern (Fig. S7). This pattern was characterized by both sharp peaks and asymmetric (short rise, long decay) waveforms in the illustrated channels (Fig. S7A, C, D). The highly stereotyped waveform of the *d*H pattern was attributed to its consistently sharp peaks and asymmetric waveforms. Similarly, we found that the *d*H pattern exhibited stronger bicoherence, skewness, and asymmetry compared to the non-*d*H pattern (Fig. S7B).

**Supplementary Figures**

**
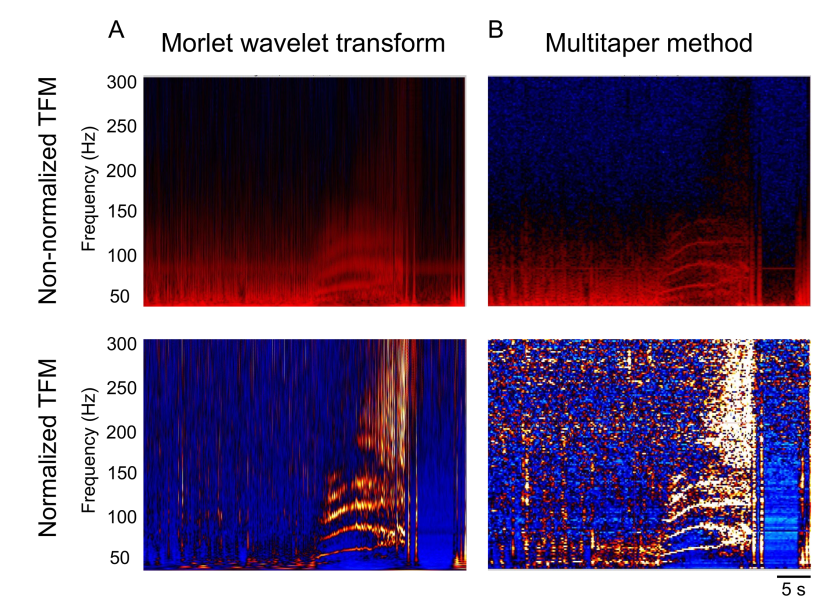
**

**Supplementary Figure 1 Illustration of non-normalized and normalized TFM using the Morlet wavelet transform and the multitaper method.** (A) Morlet wavelet transform: non-normalized TFM (top) and normalized TFM (bottom). (B) Multitaper method: non-normalized TFM (top) and normalized TFM (bottom). TFM: time-frequency maps.


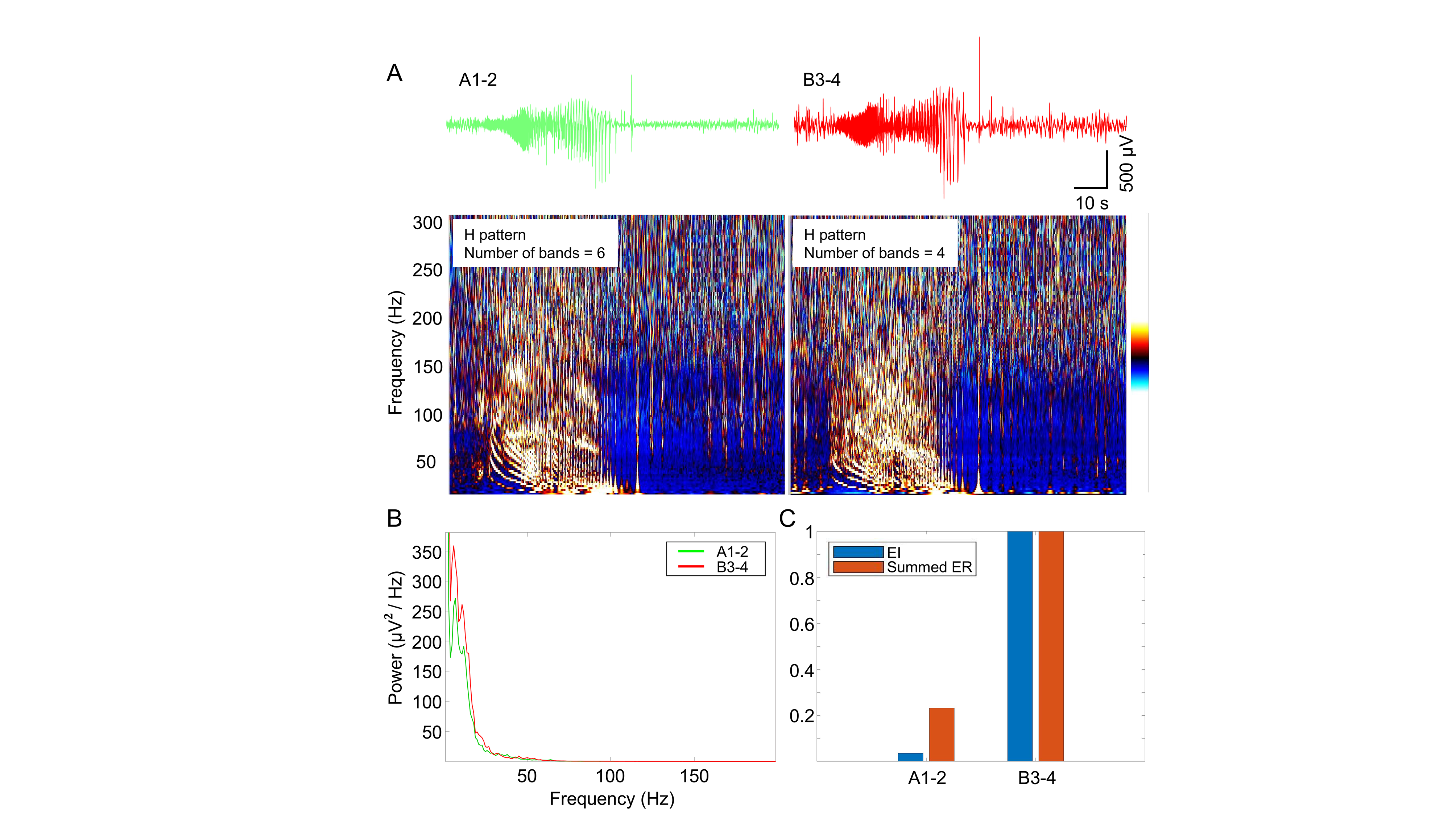


**Supplementary Figure 2 Representative illustration of the relationship between ictal discharge power and harmonic patterns.** **(A)** EEG recording (top) and corresponding time-frequency map (bottom) during a seizure. **(B)** Corresponding power spectral density. **(C)** Corresponding epileptogenicity index (EI) analysis. The EI ranks brain structures according to their spectral signature - defined as the energy ratio (ER) of high-frequency (β, γ) to low-frequency (θ, α) SEEG activity (orange-red bars) - and their delay of involvement at seizure onset. Normalized EI values (blue bars) range from 0 to 1, with 1 indicating the most epileptogenic region.

Note: Despite having lower ictal discharge power and EI values, A1-2 exhibited a higher number of H pattern bands than B3-4.


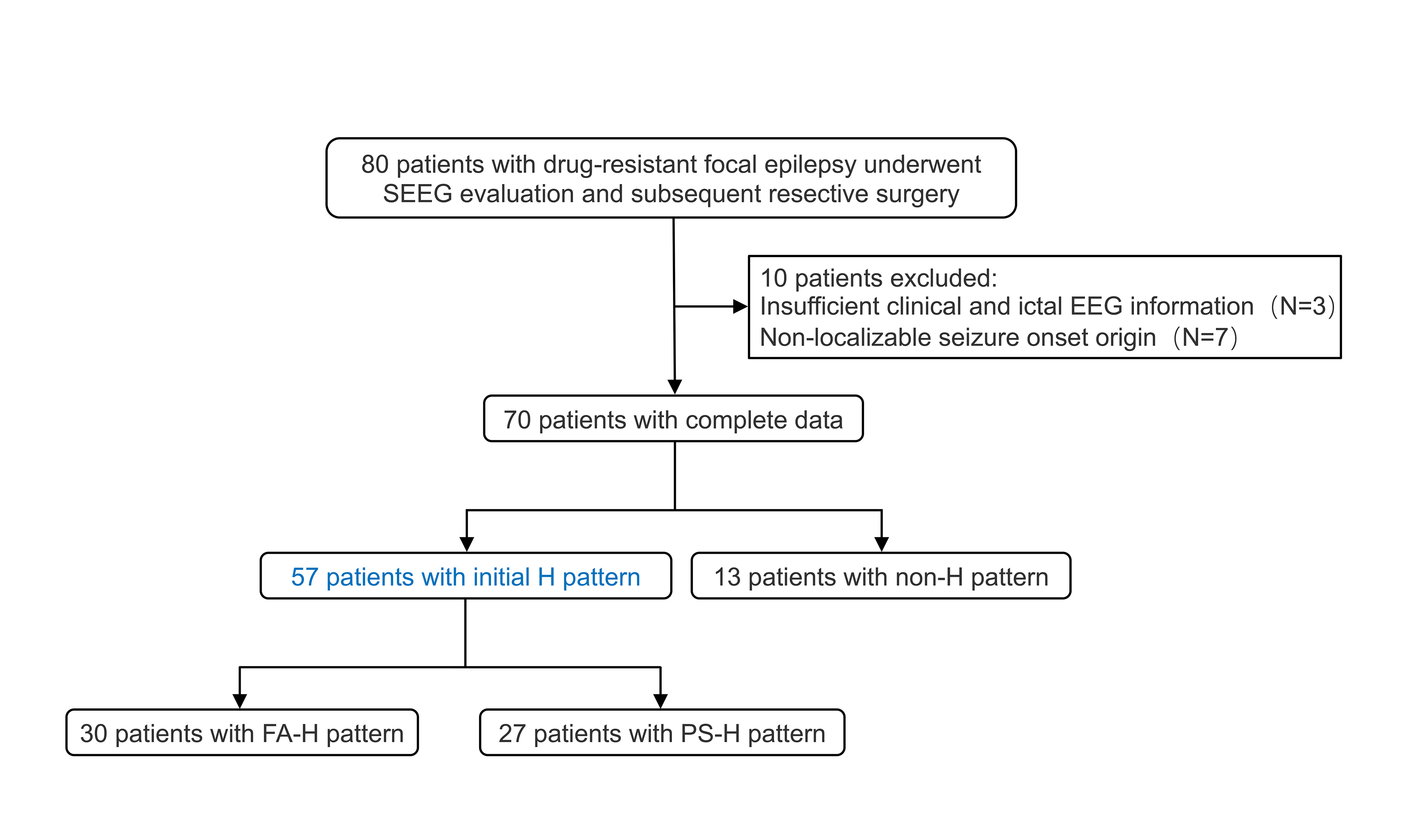


**Supplementary Figure 3 Flow chart showing the inclusion and exclusion process of patients in our study.**


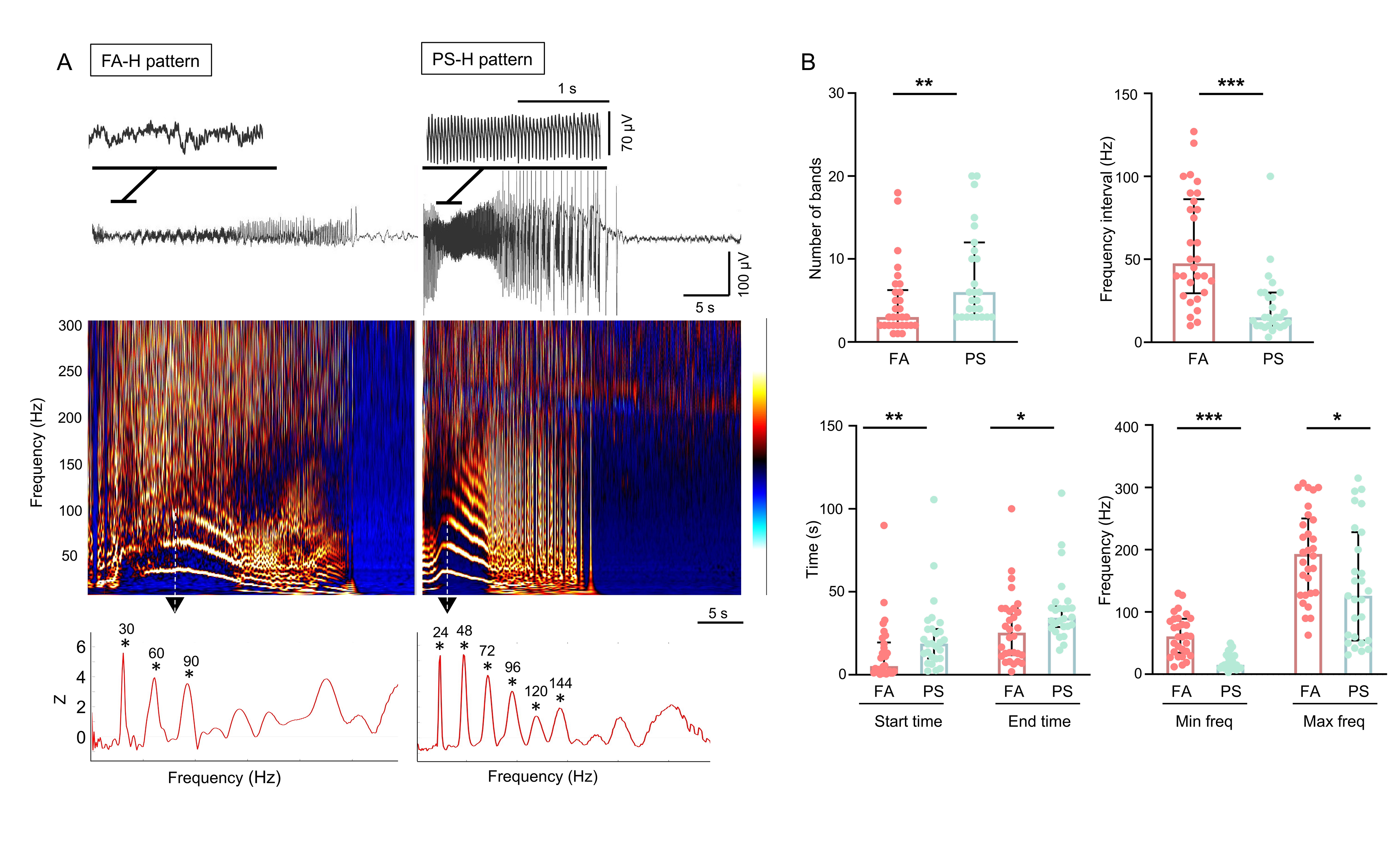


**Supplementary Figure 4 Two types of EEG segments harboring H pattern. (A)** H pattern is presented in fast activity (FA-H pattern) and irregular polyspikes (PS-H pattern) (top and middle). The power spectral density (bottom) at the maximal frequency point shows an equidistant distribution of the frequency bands. **(B)** Comparison of parameters between the FA-H pattern and PS-H pattern. The former showed a fewer number of frequency bands (3 (2-6.25) vs. 6 (3-11.5), *P* = 0.006), higher frequency interval (47.5 (29.5-86.25) vs. 15 (10-30) Hz, *P* < 0.0001), earlier start (time zero, EEG onset time; 5.2 (1.31-19.51) vs. 18.70 (10.32-27.52) s, *P* = 0.004) and end (25.4 (11.74-40) vs. 33.9 (28.59-40.25) s, *P* = 0.015) time, and higher minimal (60 (34.50-89.18) vs. 15.00(10-30) Hz, *P* < 0.0001) and maximal (193.00 (129.25-250) vs. 126.00 (55-214) Hz, *P* = 0.021) frequencies than the latter. **P* < 0.05; ***P* < 0.01; ****P* < 0.001. Max freq: maximal frequency; Min freq: minimal frequency. Statistical analysis was performed using the nonparametric Mann-Whitney U test.


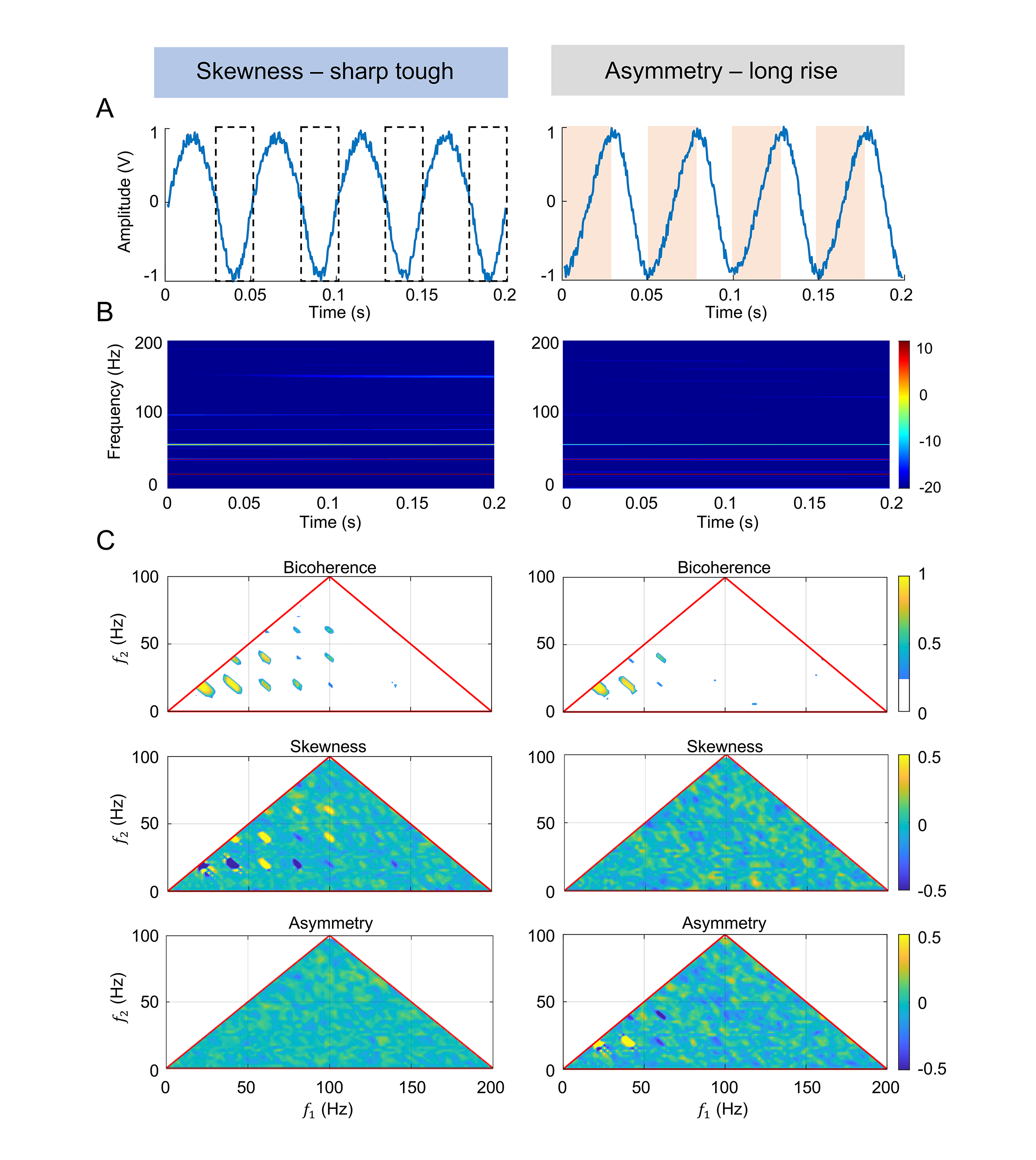


**Supplementary Figure 5 Two simulated waves to validate harmonic components induced by waveform distortion. (A)** Left: To illustrate the waveform skewness that generates the H pattern, we constructed a sinusoidal wave with T_trough_ = 0.02 s and T_peak_= 0.03 s, resulting in a wave with a primary period T= 0.05 s. Right: To demonstrate the asymmetric waveform that generates the H pattern, we constructed a sinusoidal wave with T_rise_ = 0.03 s and T_decay_ = 0.02 s, resulting in an asymmetric waveform with a primary period T= 0.05 s. **(B)** TFM for the two simulated waves. **(C)** Bispectral analysis for the two simulated waves. TFM: time frequency map.


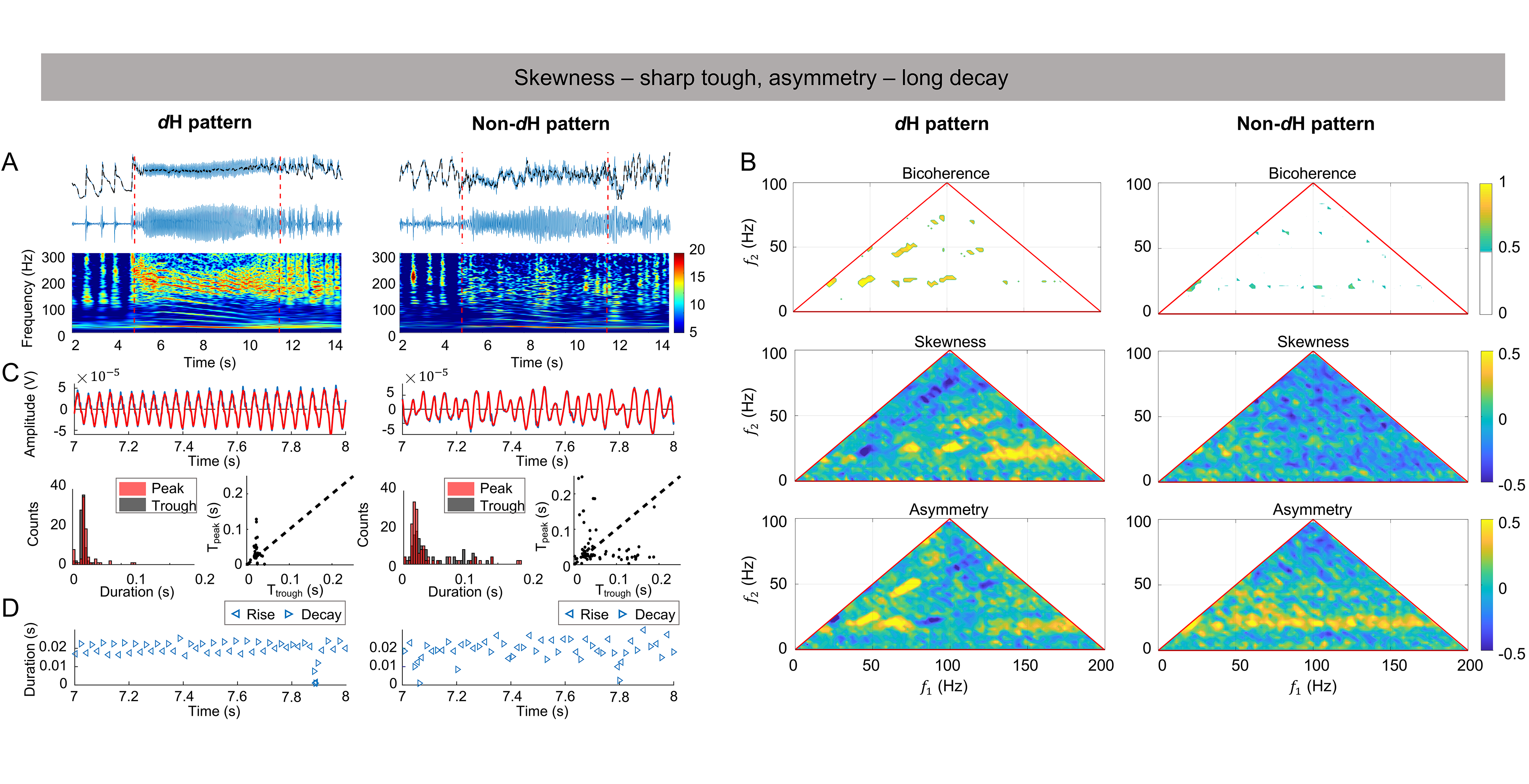


**Supplementary Figure 6 The *d*H pattern attributed to stronger skewness and asymmetry of the FA waves. (A)** Original SEEG signals in the *d*H/non-*d*H patterns (top), detrended SEEG signals (middle), TFM (bottom). **(B)** Bispectral analysis for the *d*H/non-*d*H patterns. **(C)** Comparison of peak and trough. Top: peaks and troughs of the FA waves (blue lines) in the *d*H/non-*d*H patterns are separately fitted by sin waves (red lines), respectively; Bottom: histogram (left) and scatter plot (right) depicting the distribution of T_peak_ vs. T_trough_ for the *d*H/non-*d*H patterns. **(D)** Comparison of rise (left triangles) and decay (right triangles).


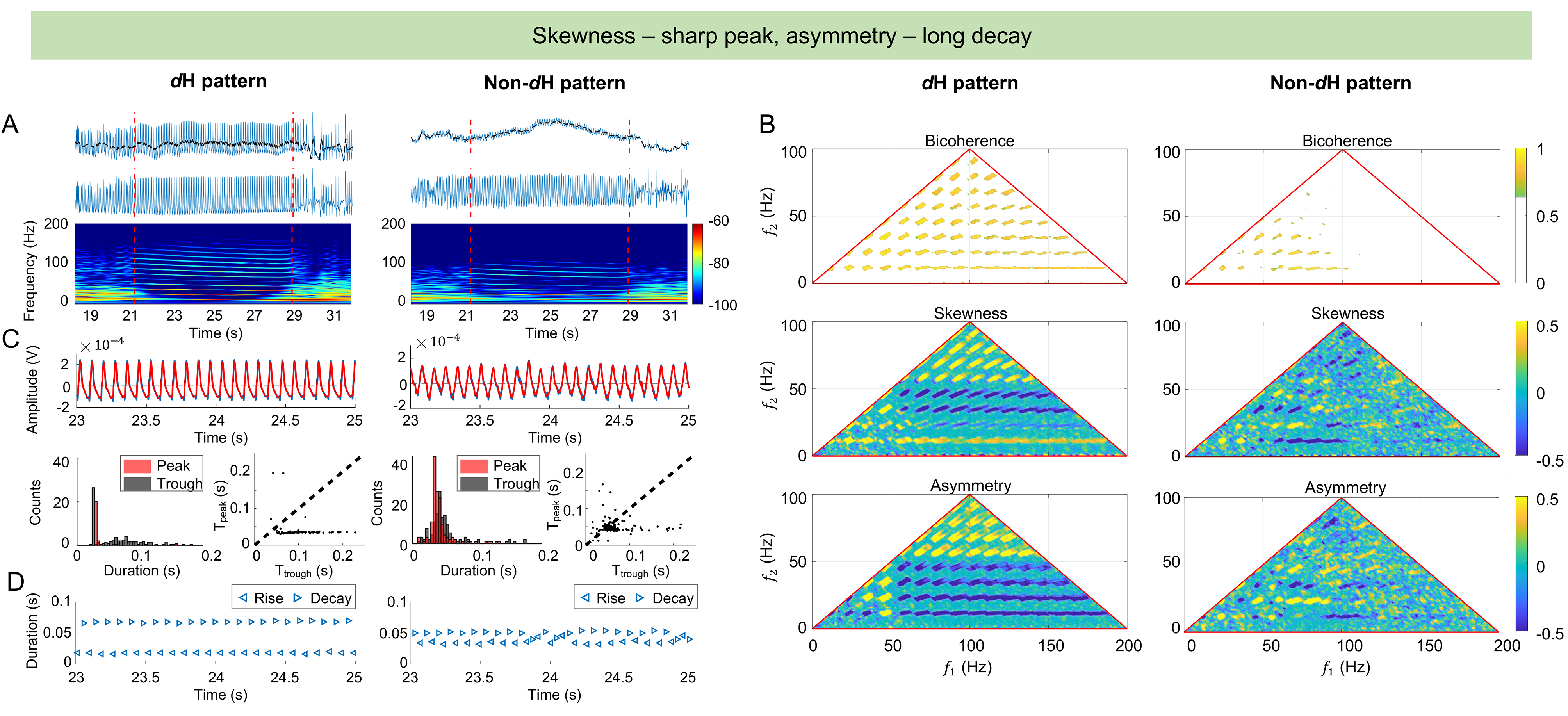


**Supplementary Figure 7 The *d*H pattern attributed to stronger skewness and asymmetry of the PS waves. (A)** Original SEEG signals in the *d*H/non-*d*H patterns (top), detrended SEEG signals (middle), and TFM (bottom). **(B)** Bispectral analysis for the *d*H/non-*d*H patterns. **(C)** Comparison of peak and trough. Top: peaks and troughs of the PS waves (blue lines) in the *d*H/non-*d*H patterns are separately fitted by sin waves (red lines), respectively; Bottom: histogram (left) and scatter plot (right) depicting the distribution of T_peak_ vs. T_trough_ for the *d*H/non-*d*H patterns. **(D)** Comparison of rise (left triangles) and decay (right triangles).


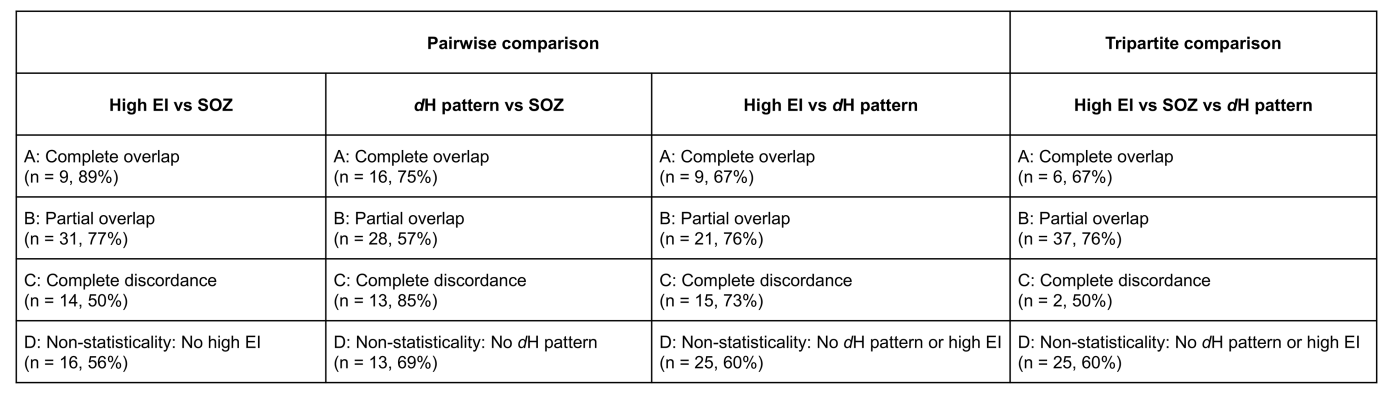


**Supplementary Figure 8: Concordance of high EI, SOZ, and *d*H pattern.** The concordance among these three markers was evaluated based on their bdEZ overlap and was categorized into four groups (A-D). The number of cases in each group and the proportion of patients with favorable outcomes were shown in parentheses.

Table S1 Comparison of contact numbers for the three markers.

| Number of contacts, n | High EI | SOZ | *d*H pattern | *P* |
| --- | --- | --- | --- | --- |
| Total | 4 (2.75-6) | 5 (3-10.25) | 4 (2-7.5) | 0.062 |
| Seizure-free group（SF） | 4 (3-6) | 5 (3-11) | 5 (2-8) | 0.379 |
| Not-seizure free group（NSF） | 4 (2-6) | 6 (3-10) | 3 (2-7) | 0.105 |
| *P*（SF vs NSF） | 0.382 | 0.604 | 0.829 |  |
