# Supplementary material for: Harmonic patterns embedded in ictal EEG signals in focal epilepsy: new insight into the epileptogenic zone
Source: BMC Med. 2026 Jan 28;24:115. doi: 10.1186/s12916-026-04665-7 (PMC12924499; doi:10.1186/s12916-026-04665-7)
Supplement: Supplementary file 4 — Additional file 4. [file 12916_2026_4665_MOESM4_ESM.docx]

Supplementary Materials for

**Harmonic patterns embedded in ictal EEG signals in focal epilepsy: new insight into the epileptogenic zone**

Lingli Hu, *et al.*

*Corresponding author: Shuang Wang. E-mail: [wangs77@zju.edu.cn](mailto:wangs77@zju.edu.cn).

Dongping Yang. E-mail: dpyang@zhejianglab.com.

**Supplementary Text**

**Supplementary Results**

**Stronger skewness and asymmetry underlying the *d*H pattern**

We presented a case with the PS-H pattern (Fig. S7). This pattern was characterized by both sharp peaks and asymmetric (short rise, long decay) waveforms (Fig. S7A, C, D). The highly stereotyped waveform of the *d*H pattern was attributed to its consistently sharp peaks and asymmetric pattern. Similarly, we found that the *d*H pattern exhibited stronger bicoherence, skewness, and asymmetry compared to the non-*d*H pattern (Fig. S7B).

**Supplementary Figures**

**
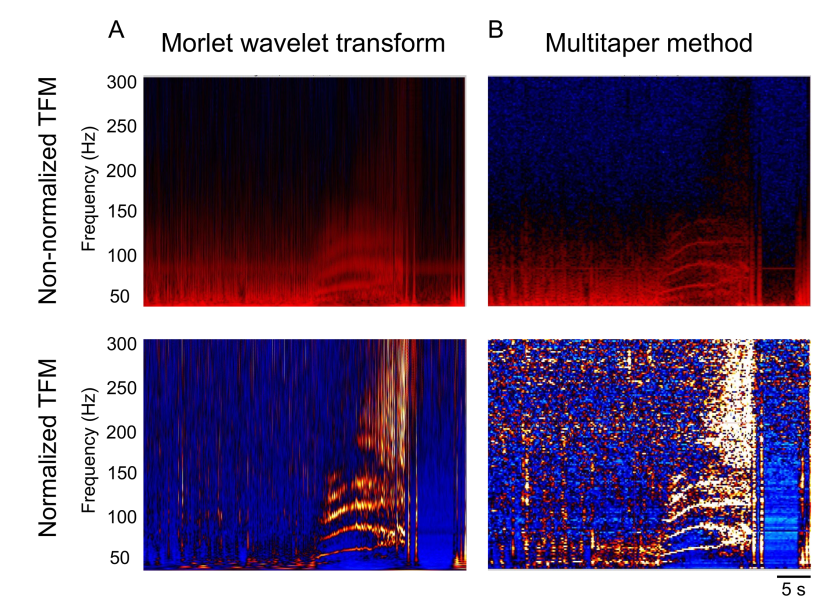
**

**Supplementary Figure 1 Illustration of non-normalized and normalized TFM using the Morlet wavelet transform and the multitaper method.** (A) Morlet wavelet transform: non-normalized TFM (top) and normalized TFM (bottom). (B) Multitaper method: non-normalized TFM (top) and normalized TFM (bottom). TFM: time-frequency maps


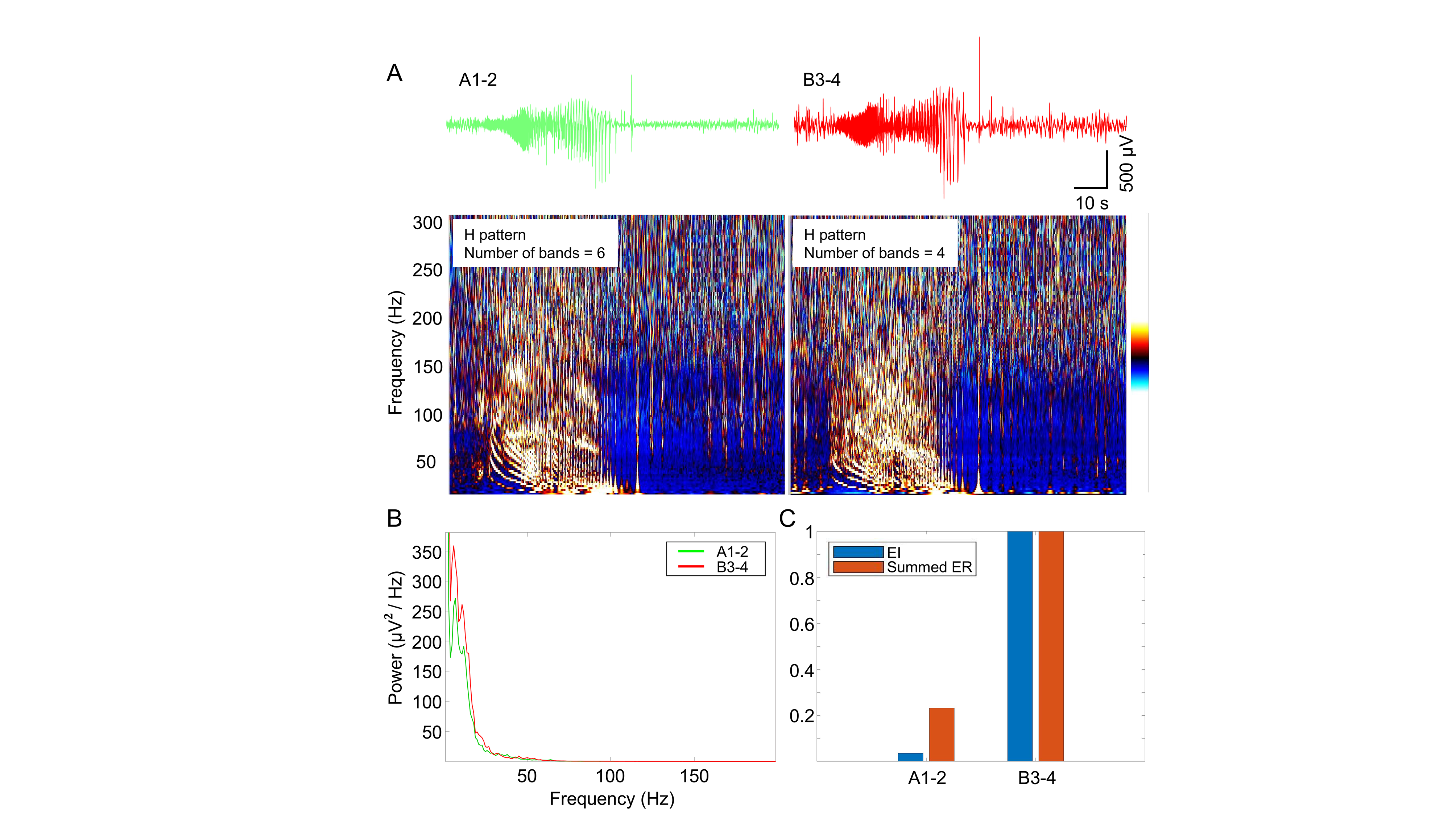


**Supplementary Figure 2 Representative illustration of the relationship between ictal discharge power and harmonic patterns.** **(A)** EEG recording (top) and corresponding time-frequency map (bottom) during a seizure. **(B)** Corresponding power spectral density. **(C)** Corresponding EI analysis. A1-2 exhibited a higher number of H pattern bands than B3-4, despite having lower ictal discharge power and EI values.


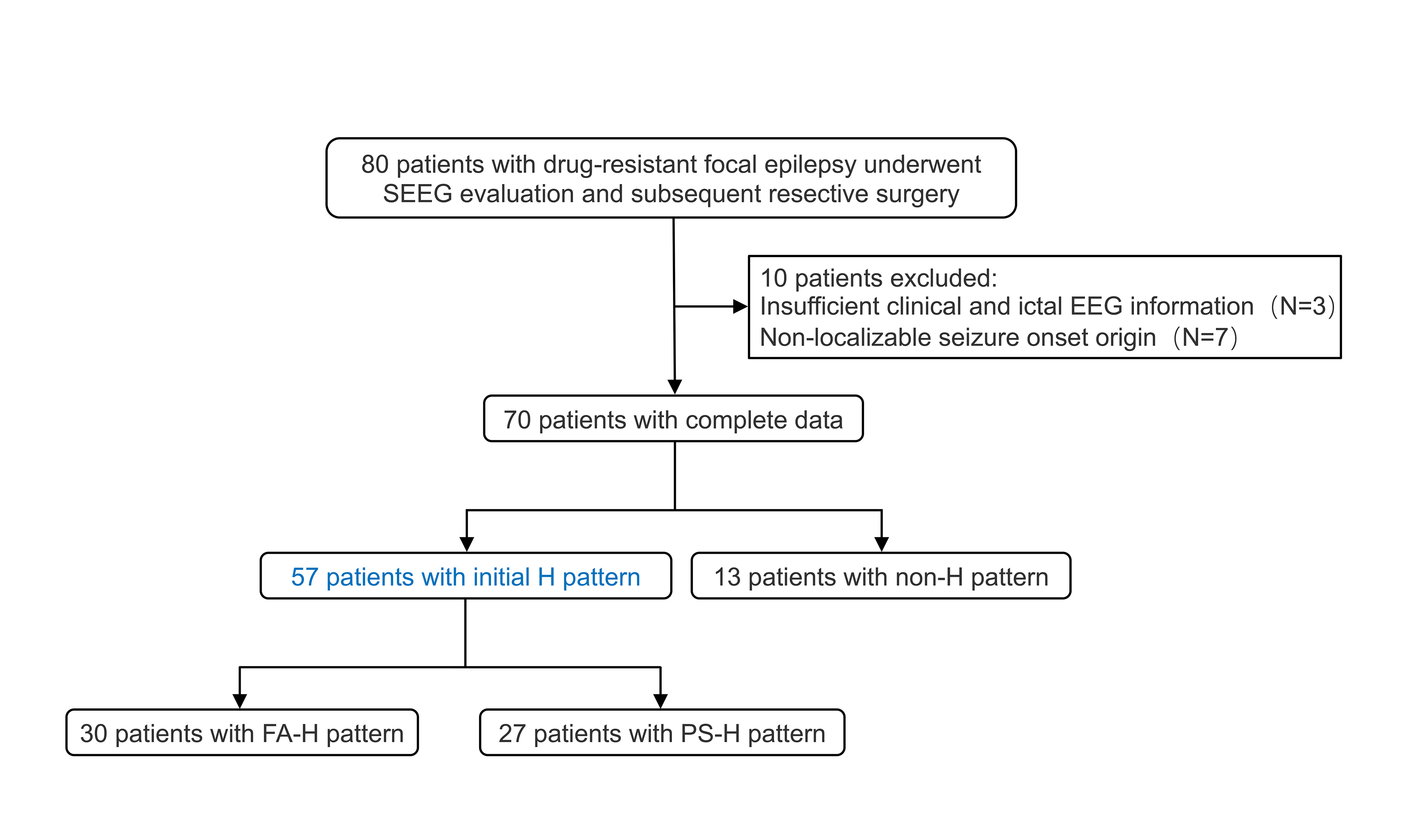


**Supplementary Figure 3 Flow chart showing the inclusion and exclusion process of patients in our study.**


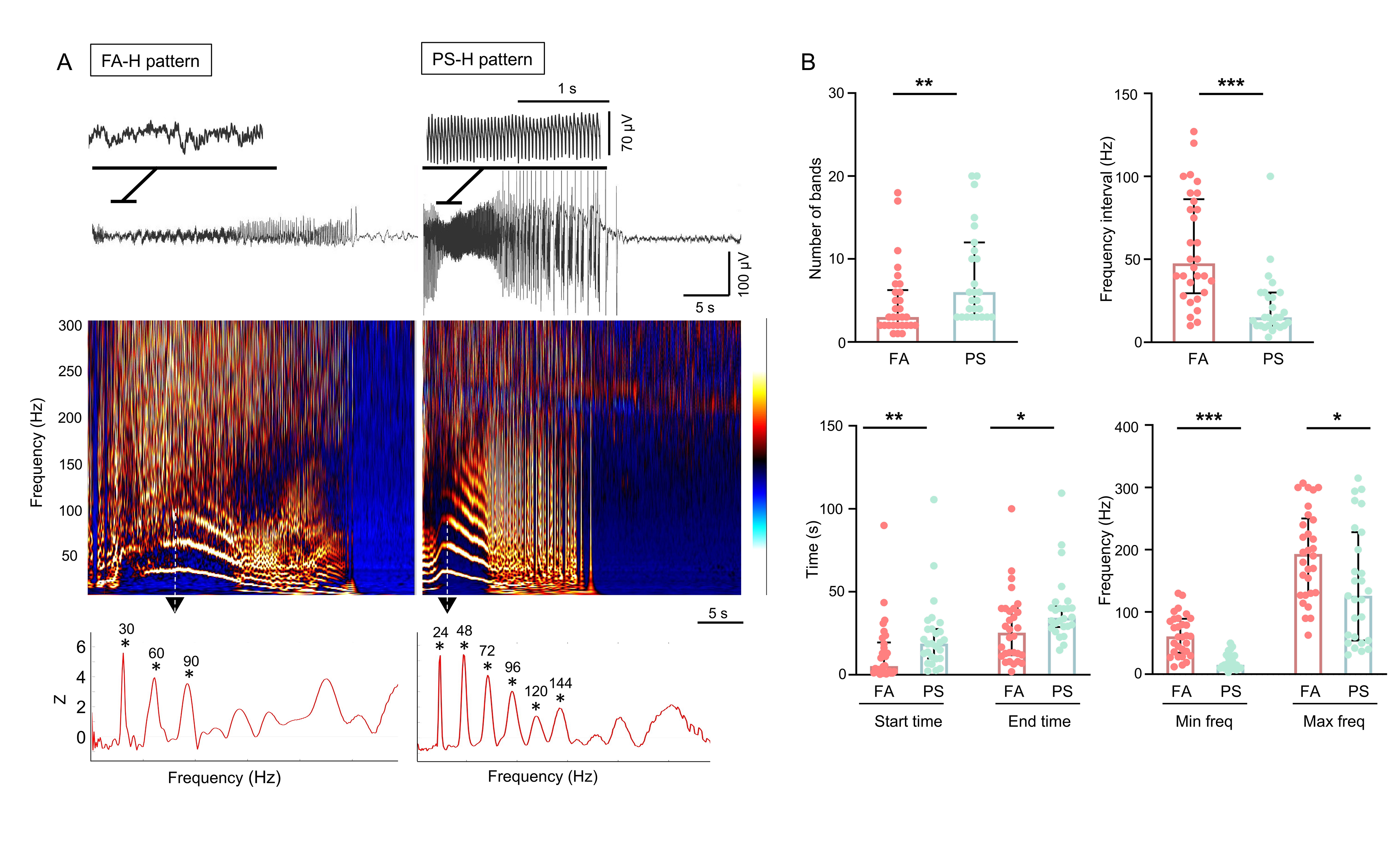


**Supplementary Figure 4 Two types of EEG segments harboring H pattern. (A)** H pattern is presented in fast activity (FA-H pattern) and irregular polyspikes (PS-H pattern) (top and middle). The power spectral density (bottom) at the maximal frequency point shows an equidistant distribution of the frequency bands. **(B)** Comparison of parameters between the FA-H pattern and PS-H pattern. The former showed a fewer number of frequency bands (3 (2-6.25) vs. 6 (3-11.5), *P* = 0.006), higher frequency interval (47.5 (29.5-86.25) vs. 15 (10-30) Hz, *P* < 0.0001), earlier start (time zero, EEG onset time; 5.2 (1.31-19.51) vs. 18.70 (10.32-27.52) s, *P* = 0.004) and end (25.4 (11.74-40) vs. 33.9 (28.59-40.25) s, *P* = 0.015) time, and higher minimal (60 (34.50-89.18) vs. 15.00(10-30) Hz, *P* < 0.0001) and maximal (193.00 (129.25-250) vs. 126.00 (55-214) Hz, *P* = 0.021) frequencies than the latter. **P* < 0.05; ***P* < 0.01; ****P* < 0.001. Max freq: maximal frequency; Min freq: minimal frequency. Statistical analysis was performed using the nonparametric Mann-Whitney U test.


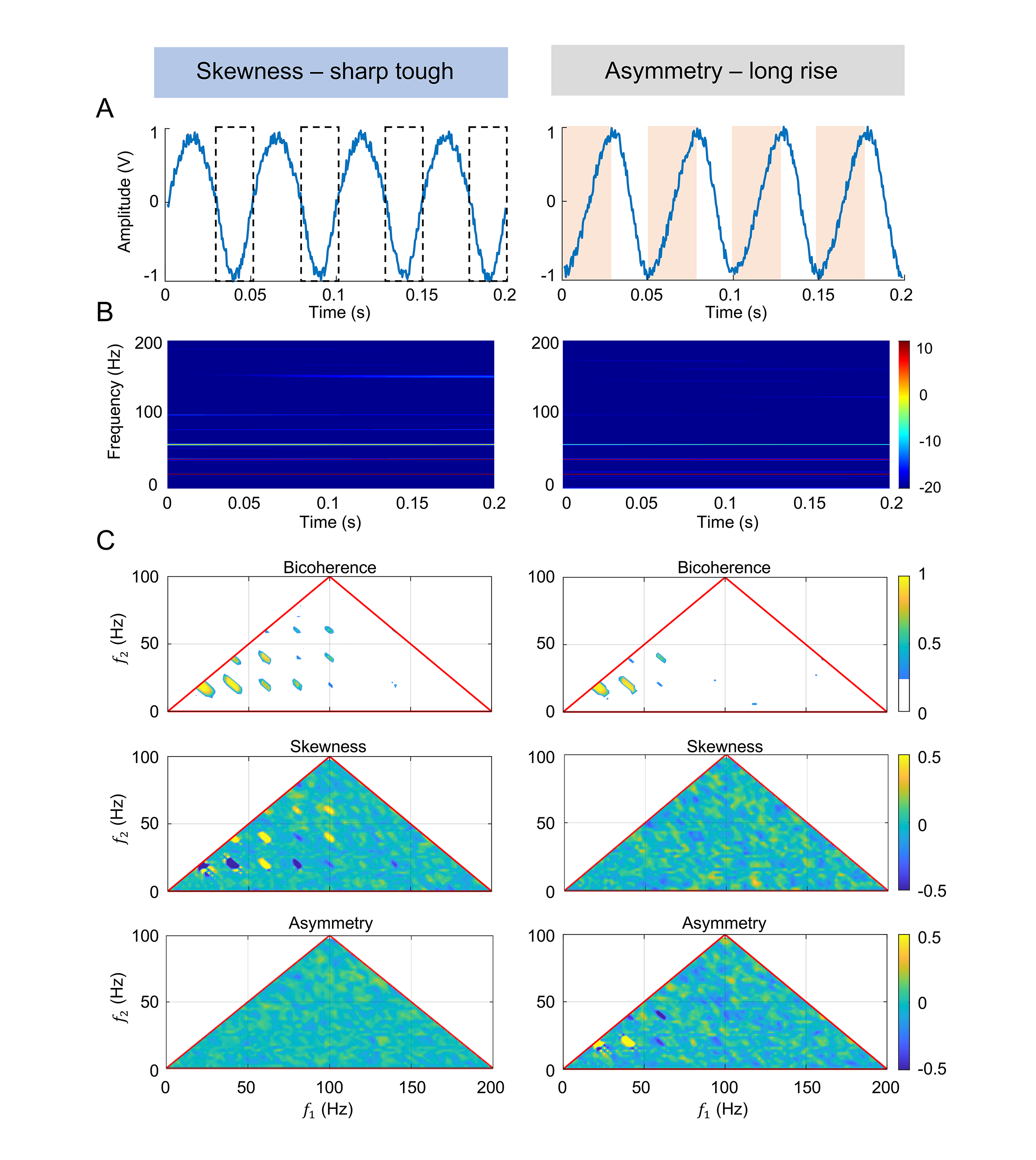


**Supplementary Figure 5 Two simulated waves to validate harmonic components induced by waveform distortion. (A)** Left: to illustrate the waveform that generates H pattern with sharp troughs, we constructed a sinusoidal wave with T_trough_ = 0.02 s and T_peak_= 0.03 s, resulting in a wave with a primary period T= 0.05 s. Right: to demonstrate the asymmetric waveform that generates H pattern, we constructed a sinusoidal wave with T_rise_ = 0.03 s and T_decay_ = 0.02 s, resulting in an asymmetric waveform with a primary period T= 0.05 s. **(B)** TFM for the two simulated waves. **(C)** Bispectral analysis for the two simulated waves. TFM: time frequency map.


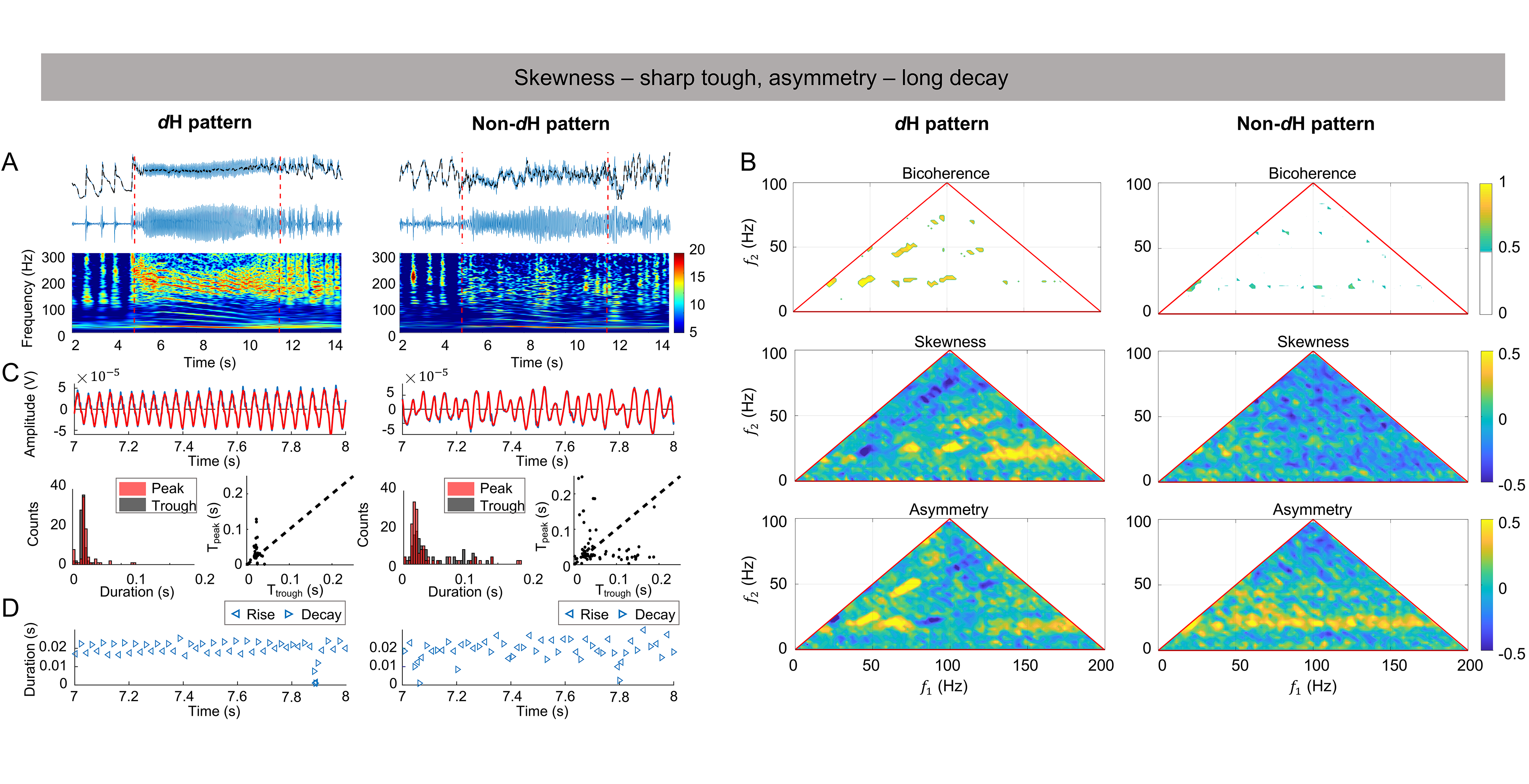


**Supplementary Figure 6 The *d*H pattern attributed to stronger skewness and asymmetry of the FA waves. (A)** Original SEEG signals in the *d*H/non-*d*H patterns (top), detrended SEEG signals (middle), TFM (bottom). **(B)** Bispectral analysis for the *d*H/non-*d*H patterns. **(C)** Comparison of peak and trough. Top: peaks and troughs of the FA waves (blue lines) in the *d*H/non-*d*H patterns are separately fitted by sin waves (red lines), respectively; Bottom: histogram (left) and scatter plot (right) depicting the distribution of T_peak_ vs. T_trough_ for the *d*H/non-*d*H patterns. **(D)** Comparison of rise (left triangles) and decay (right triangles).


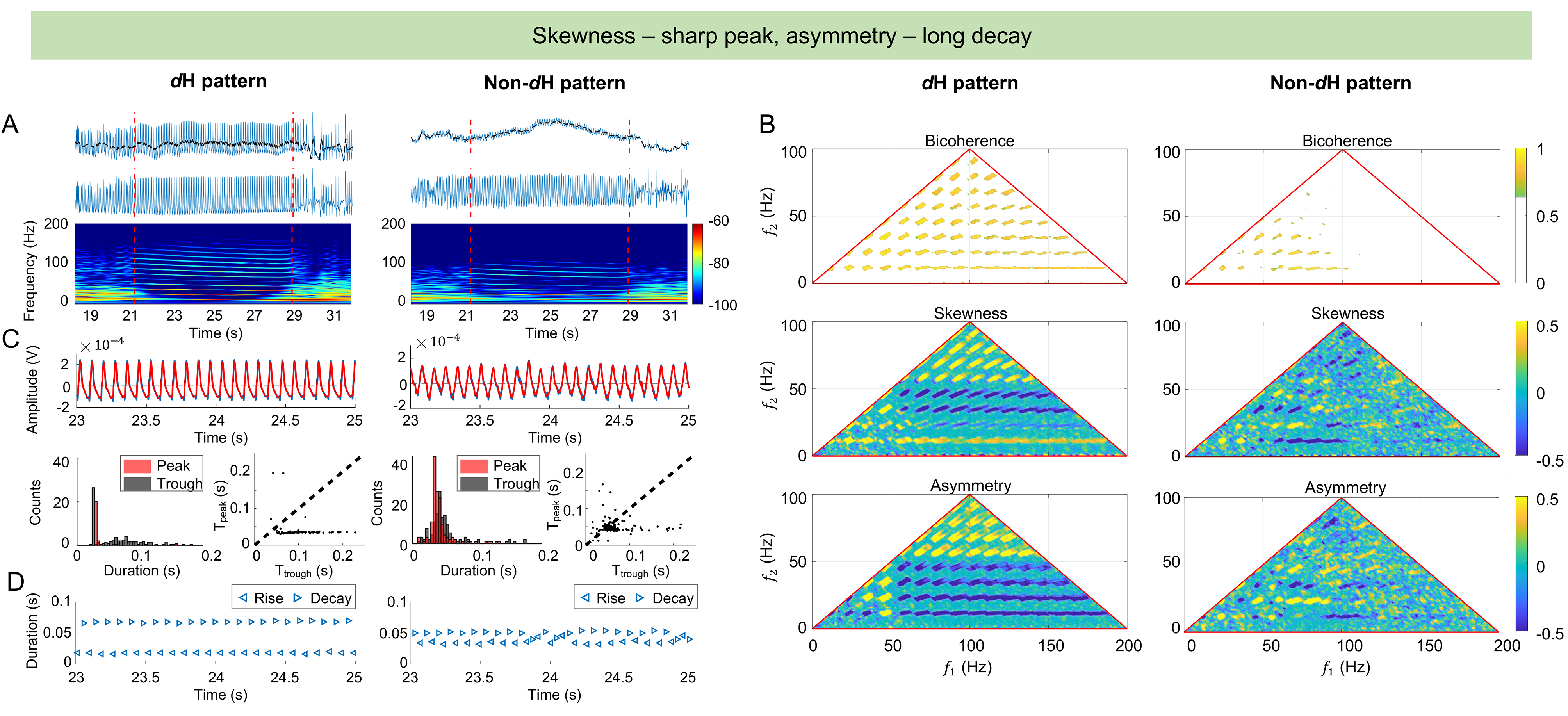


**Supplementary Figure 7 The *d*H pattern attributed to stronger skewness and asymmetry of the PS waves. (A)** Original SEEG signals in the *d*H/non-*d*H patterns (top), detrended SEEG signals (middle), and TFM (bottom). **(B)** Bispectral analysis for the *d*H/non-*d*H patterns. **(C)** Comparison of peak and trough. Top: peaks and troughs of the PS waves (blue lines) in the *d*H/non-*d*H patterns are separately fitted by sin waves (red lines), respectively; Bottom: histogram (left) and scatter plot (right) depicting the distribution of T_peak_ vs. T_trough_ for the *d*H/non-*d*H patterns. **(D)** Comparison of rise (left triangles) and decay (right triangles).


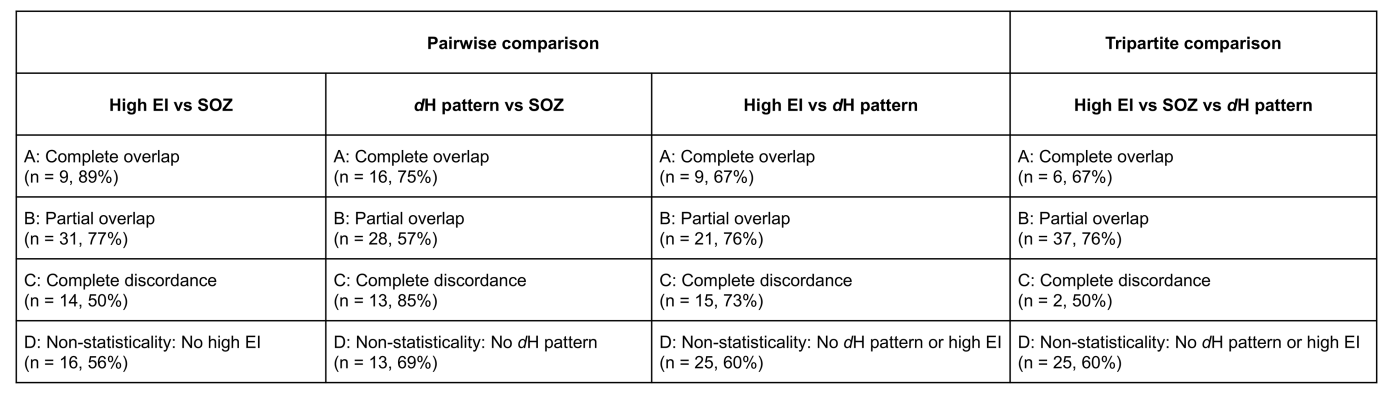


**Supplementary Figure 8: Concordance of high EI, SOZ, and *d*H pattern.** The concordance among these three markers was evaluated based on their bdEZ overlap and was categorized into four groups (A-D). The number of cases in each group and the proportion of patients with favorable outcomes were shown in parentheses.

Table S1 Comparison of contact numbers for the three markers.

| Number of contacts, n | High EI | SOZ | *d*H pattern | *P* |
| --- | --- | --- | --- | --- |
| Total | 4 (2.75-6) | 5 (3-10.25) | 4 (2-7.5) | 0.062 |
| Seizure-free group（SF） | 4 (3-6) | 5 (3-11) | 5 (2-8) | 0.379 |
| Not-seizure free group（NSF） | 4 (2-6) | 6 (3-10) | 3 (2-7) | 0.105 |
| *P*（SF vs NSF） | 0.382 | 0.604 | 0.829 |  |
